# Muscle Traits, Sarcopenia, and Sarcopenic Obesity: A Vitamin D Mendelian Randomization Study

**DOI:** 10.3390/nu15122703

**Published:** 2023-06-09

**Authors:** Joshua P. Sutherland, Ang Zhou, Elina Hyppönen

**Affiliations:** 1 Australian Centre for Precision Health, Unit of Clinical and Health Sciences, University of South Australia, Adelaide 5000, Australia; joshua.sutherland@mymail.unisa.edu.au (J.P.S.); ang.zhou@unisa.edu.au (A.Z.); 2 South Australian Health and Medical Research Institute, Adelaide 5000, Australia; 3 Medical Research Council Biostatistics Unit, University of Cambridge, Cambridge CB2 0SR, UK

**Keywords:** vitamin D, skeletal muscle, grip strength, sarcopenia, sarcopenic obesity, vitamin D deficiency, nutritional status, public health

## Abstract

(1) Background: Observational studies associate vitamin D deficiency with muscle disorders, while some clinical trial data support a minor association between the vitamin and skeletal muscle performance in healthy subjects. Vitamin D receptor knockout mice studies confirm the relationship between vitamin D and skeletal muscle; however, causal inference in humans is challenging due to the ethical implications of including vitamin D-deficient participants in randomized trials. This study uses genetic methods to safely explore causal underpinnings for the relationship between 25(OH)D concentrations and skeletal muscle-related traits, including grip strength and combined arm skeletal muscle mass, and extends this analysis to suspected pathophysiology in the form of probable sarcopenia and sarcopenic obesity. (2) Methods: We conducted Mendelian randomization (MR) analyses in up to 307,281 participants from the UK Biobank of whom 25,414 had probable sarcopenia and 16,520 had sarcopenic obesity. In total, 35 variants were used to instrument 25(OH)D and MR analyses conducted using multiple approaches. (3) Results: Genetic analyses provided support for a relationship between genetically predicted higher 25(OH)D and skeletal muscle traits, with linear MR analyses for grip strength showing 0.11 kg (95% CI 0.04, 0.19) greater contractile force per 10 unit higher 25(OH)D, while there was a modest association with skeletal muscle mass (0.01 kg (95% CI 0.003, 0.02) greater muscle mass). For probable sarcopenia risk, there was suggestive evidence for lower odds by higher 25(OH)D (OR 0.96 (95% CI 0.92, 1.00)); however, this did not reflect an association with sarcopenic obesity (OR 0.97 (95% CI 0.93, 1.02)), but was seen in probable sarcopenia cases who were not obese (OR 0.92 (95% CI 0.86, 0.98)). Results were similar across multiple MR approaches. (4) Conclusions: Our study supports a causal relationship between 25(OH)D and skeletal muscle health. While evidence for benefit did not extend to lower risk of sarcopenic obesity, effective vitamin D-deficiency prevention strategies may help reduce age-related muscle weakness.

## 1. Introduction

Vitamin D is a crucial component in the regulation of calcium homeostasis and bone metabolism; however, evolving research demonstrates wide ranging roles for the vitamin in other tissues, including skeletal muscle tissue [1]. Interest in vitamin D and skeletal muscle tissue has arisen from observations of myopathy and hypotonia concurring with rickets and osteomalacia, which are diseases remediated by reversing severe vitamin D deficiency [2] (25-hydroxy vitamin D (25(OH)D) < 25 nmol/L [3]). Several decades of observational studies have supported an association between vitamin D deficiency and impaired skeletal muscle health, including sarcopenia and sarcopenic obesity [4,5,6,7], which are common concerns in the elderly, reflecting age-related loss of skeletal muscle integrity. Experiments using the myocyte-specific vitamin D receptor (VDR) knockout mouse mice [8,9] provide further support for the role of vitamin D-related signalling pathways in skeletal muscle development and in determining lean mass and muscle function, highlighting biological relevance and the need for research in this area.

Clinical supplementation trials, aimed at optimizing 25(OH)D, have supported minor strength improvements in healthy athletes [10]; however, vitamin D supplementation trials for sarcopenia have presented conflicting results [6], with a subset of evidence suggesting co-administration with supplemental protein may have particular benefit for improving muscle strength [7]. Crucially, vitamin D supplementation trials often lack inclusion of severely deficient participants due to ethical concerns or recruitment challenges [11], making it difficult to use non-representative and low-powered clinical trial data to facilitate causal inference in this area. There is additionally a notable lack of clinical data examining the effects of supplemental vitamin D in those with sarcopenic obesity [7].

A further line of evidence can be obtained from studies using Mendelian randomization (MR), which uses genetic variants that approximate the exposure as an instrumental variable or “proxy indicator” and can provide evidence of causal effects in situations where it is unethical or infeasible to conduct RCTs [12]. Observational research with vitamin D and skeletal muscle tissue has confounding challenges, such as the association between outdoor time (i.e., vitamin D stimulating UVB exposure) and increased physical activity [13] (i.e., with physical activity having independent effect on myocyte expression), while paradoxical observations can arise due to skeletal muscle tissue itself acting as a 25(OH)D storage site [14]. The MR can largely overcome these challenges. To date, there are several MR studies that have looked into the association between vitamin D and muscle traits (grip strength [15,16]—a tool often used to provide rapid indication of an individual’s general muscle strength [17]—and skeletal muscle mass [18]), while evidence for association with skeletal muscle pathology (i.e., sarcopenia) is inconclusive [19] with only one multi-exposure MR study having used a comparatively low powered 25(OH)D instrument. These prior muscle trait studies provide promising support for an association between higher 25(OH)D and general muscle health; however, the role of 25(OH)D in sarcopenia warrants a more conclusive investigation, with an adjoining focus on sarcopenic obesity. In this study we use information from up to 307,281 UK Biobank participants to examine evidence for a causal role of 25(OH)D in skeletal muscle health. Importantly, in addition to grip strength and muscle mass, we include exploration of pathophysiology, as reflected by associations with probable sarcopenia [20,21] and sarcopenic obesity.

## 2. Methods

The UK Biobank is a large-scale, prospective cohort comprising a baseline assessment of 502,316 individuals aged 37–73 years, undertaken from March 2006 and July 2010 in England, Scotland, and Wales [22]. Volunteer participants partook in questionnaire surveys, underwent physical assessments, and provided biological samples for biomarker and genetic assays. Primary analyses in our study were limited to unrelated participants of European ancestry, who had measures for serum 25(OH)D concentrations and available data for the muscle trait outcomes (*n* = 307,281); these factors and information pertaining to missing data are outlined in Figure S1 [23].

### 2.1. Grip Strength, Muscle Mass, and Probable Sarcopenia

Grip strength assessment and bioimpedance analysis of skeletal muscle mass and whole-body fat mass were performed at the initial baseline assessment centre visit and used a Jamar J00105 hydraulic hand dynamometer and Tanita BC418MA body composition analyser (in conjunction with inputted height data, acquired from a Seca 202 stadiometer), respectively. For our grip strength analyses, we used the left-and-right-hand average contractile force, measured in kilograms (kg). We use a previously developed definition for probable sarcopenia [20,21], and defined cases as those participants who had grip strength <27 kg of force (males) and <16 kg of force (females). Whole-body fat mass was used to calculate body fat percentage (whole-body fat mass divided by body weight and multiplied by 100) and the conventional cut-off points of ≥25% for males and ≥35% for females were used to determine obesity [24]. Analyses on probable sarcopenic obesity and non-obesity probable sarcopenia used participants without sarcopenia as the control group. Analysis of lean skeletal muscle mass used combined left-and-right-arm data.

### 2.2. Measured and Genetically Instrumented 25(OH)D

The LIAISON XL 25(OH)D assay (DiaSorin, Stillwater, OK, USA) was used to measure baseline serum 25(OH)D concentrations, as described in the Supplementary Materials [23]. Covariates used in adjustments were acquired at baseline from self-reported, touchscreen questionaries (age, sex, physical activity, smoking, alcohol, birth location), residential address data (Townsend deprivation index) [25], physical assessments (height and waist circumference), with these and additional covariates (assessment centre, month of 25(OH)D acquisition, genetic-related information, and 25(OH)D measurement nuisance factors) outlined in full in the Supplementary Materials (pp. 3–4) [23]. We constructed a weighted genetic score by assembling 35 common autosomal SNPs that were discovered from a genome-wide association analysis on measured 25(OH)D concentration [26,27] and replicated in the SUNLIGHT consortium [28]. The construction of the vitamin D genetic score, and the selection of the variants used, are described in the Supplementary Materials (p. 5) [23]. Ethics approval for the UK Biobank was granted by the National Information Governance Board for Health and Social Care and North West Multicentre Research Ethics Committee (11/NW/0382). The present analysis operates under UK Biobank application 20175.

### 2.3. Statistical Methods

The main phenotypic analyses were conducted using linear and logistic regression for continuous (grip strength and skeletal muscle mass) and binary outcomes (probable sarcopenia, and probable sarcopenia with or without obesity), respectively. We fitted two models, a simple and a full model; in the simple model, we adjusted for study structure factors—age, sex, height, and assessment centre—as well as nuisance factors, which could affect the measured 25(OH)D concentrations (blood sampling month, fasting time before sample acquisition, and aliquot); in the full model, we further adjusted for lifestyle factors, including waist circumference, physical activity (low, moderate, and high), alcohol (daily or almost daily, once or twice a week, three of four times a week, one to three times a month, special occasions only, and never), smoking (non-smokers, former smokers, and current smokers), education (none, national vocational qualification/certificate of secondary education/A-levels, and degree/professional), and Townsend deprivation index (quartile division of index: least to most deprivation). Height and waist circumference were chosen for phenotypic adjustments—to account for body shape and obesity—rather than using body mass index (BMI), due to BMI’s calculation explicitly including measure of skeletal muscle mass via body weight. To examine the shape of the association between 25(OH)D and the muscle trait outcomes, we used a likelihood ratio test and compared model fit between the best-fitting fractional polynomial model and the linear model [29].

We performed linear MR analyses to examine genetic evidence for causality in the association of measured 25(OH)D concentrations and muscle traits, including grip strength, arm skeletal muscle mass, probable sarcopenia, and probable sarcopenia with or without obesity (Supplementary Materials, pp. 6–7 and Figures S2 and S3) [23], with the genetic score-based one-sample MR analyses presented as primary findings. We also examined the presence of non-linearity of association using two available MR methods [30,31] (Supplementary Materials, pp. 8–9 and Figure S3) [23]. Linear and non-linear analyses are detailed in the Supplementary Materials [23]. As our analysis included three (correlated) outcomes, to ensure robust evidence of association, we applied Bonferroni correction to control type I error rate, taking a *p*-value below 0.05/3 = 0.02 as evidence for an association.

Valid causal inference in MR analysis is reliant on three key assumptions [32] (Figure S2) [23]; in the context of our study, they can be outlined as the following: (1) the vitamin D genetic score associates with measured 25(OH)D concentrations; (2) the vitamin D genetic score has no direct effect on muscle traits; (3) the vitamin D genetic score does not associate with confounders of measured 25(OH)D and muscle traits. In relation to the first MR assumption, the association of the vitamin D genetic score was examined with 25(OH)D concentrations in the UK Biobank (Figure S4) [23]. Horizontal pleiotropy is a phenomenon wherein the genetic instrument associates with outcomes via pathways other than the exposure of interest; if present, it will violate the second and/or third MR assumption and will resultantly bias the analyses. We implemented the following strategies to evaluate any potential horizontal pleiotropy: (i) We assessed the association of the vitamin D genetic score with a series of potential confounders in the UK Biobank, which included the Townsend deprivation index, body mass index, alcohol intake, smoking, physical activity, and education (Table S1 and Figure S5) [23]. (ii) We assigned variants to four functional blocks (renal, blood, lipids/metabolic, and unclassified) and conducted a leave-block-out analysis, designed to assess if our findings were driven by any specific blocks of potentially pleiotropic variants (in particular pleiotropy by lipid traits) [33] (Supplementary Materials, p. 8, Tables S2 and S3) [23]. (iii) In the MR analyses, we employed five sensitivity analyses, including IVW, MR-Egger, Weighted Median MR, Weighted Mode MR, and MR-PRESSO (Supplementary Materials, pp. 6–7) [23] with relatively independent assumptions on pleiotropy.

R version 3.6.1 was used for non-linear MR (‘nlmr’ and ‘SUMnlmr’ packages) and linear MR sensitivity analyses (‘TwoSampleMR’ and ‘MRPRESSO’ packages), and STATA version 14.1 (Stata-Corp LP, College Station, TX, USA) was used for all other analyses.

## 3. Results

Table 1 shows baseline participant characteristics and distribution of 25(OH)D, 25(OH)D < 25 nmol/L, grip strength, probable sarcopenia cases, sarcopenic obesity cases, and arm skeletal muscle mass. The average 25(OH)D concentration was 49.82 nmol/L, with 11.70% of the participants (*n* = 36,009) having 10–24.9 nmol/L. Concentrations for average 25(OH)D were higher in participants living in the southern-most latitudinal zone, those with less socioeconomic deprivation, non-smokers, lowest BMIs, and those with higher levels of physical activity. Grip strength was higher in those with the greatest level of physical activity, educational status, and the lowest social deprivation. Arm skeletal muscle mass was higher in males and in those <60 years of age. At baseline, 8.28% (*n* = 25,414) had probable sarcopenia, with an overrepresentation of females and those over 60 years of age (10% and 12%, respectively), as well as of those with the highest level of BMI, social deprivation, and sedentary behaviour. Sarcopenic obesity was notably more common in women compared to men, those aged ≥60 years vs. <60, and those with less compared to more physical activity. Table S4 [23] additionally presents data for height and waist circumference; similar trends were seen between BMI and waist circumference, while height differences had notable impact on skeletal muscle mass (greater mass in the tallest participants) and probable sarcopenia (greater prevalence in the shorter participants). Figure S6 [23] shows positive probable sarcopenia cases to have 0.59 kg less of arm skeletal muscle mass, relative to negative cases.

### 3.1. Grip Strength

**Figure 1:**
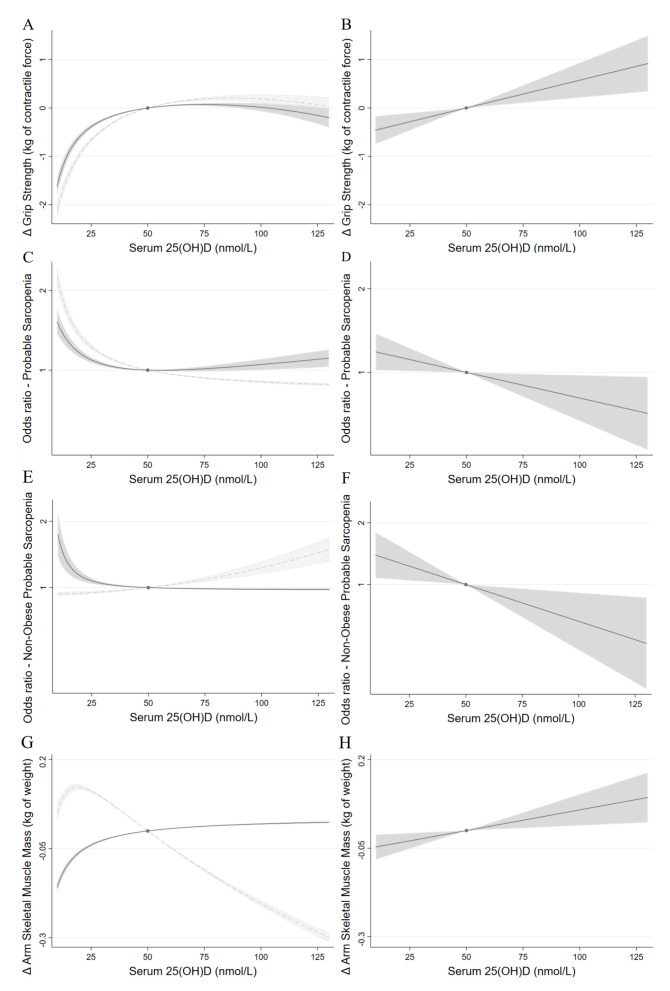
Phenotypic (**A**,**C**,**E**,**G**) and genetic (**B**,**D**,**F**,**H**) associations of 25(OH)D with grip strength (**A**,**B**), probable sarcopenia (**C**,**D**), non-obese probable sarcopenia (**E**,**F**), and arm skeletal muscle mass (**G**,**H**) in the UK Biobank, with genetic associations (**B**,**D**,**F**,**H**) projected on the measured 25(OH)D scale. Shaded areas reflect 95% confidence intervals, and the dot represents the reference point of 50 nmol/L. (Genetic analysis: **B**,**D**,**F**,**H**): the x axis—noted at serum 25(OH)D—refers to the measured 25(OH)D scale upon which the genetic analysis has been mapped. (Phenotypic analysis: **A**,**C**,**E**,**G**): simple models (light grey dashed line) were adjusted for sex, age, height, assessment centre, and nuisance factors that could affect serum 25(OH)D measurements, including month in which blood sample was taken, fasting time before blood sample was taken, and sample aliquots for measurement, with full models (dark grey solid line) additionally adjusted for educational status, the Townsend depravation index, waist circumference, physical activity, alcohol, and smoking. (Genetic analysis: **B**,**D**,**F**,**H**): adjusted for age, sex, assessment centre, birth locations, SNP array, top 40 genetic principal components, and nuisance factors that could affect serum 25(OH)D measurements, including month in which blood sample was taken, fasting time before blood sample was taken, and sample aliquots for measurement. The notable change in the phenotypic association with skeletal muscle mass by confounder adjustment was largely due to the inclusion of waist circumference, which was strongly correlated with both 25(OH)D and muscle mass (r = −0.1609, r = 0.689, respectively; *p* < 0.1 × 10^–4^ for both).

In our phenotypic analyses, we observed a non-linear inverse relationship between measured 25(OH)D and grip strength (Figure 1A), with both the simple and fully adjusted models following similarly in shape. Grip strength decreased precipitously below 25 nmol/L 25(OH)D, with the adjusted association appearing to plateau from 50 nmol/L onwards. For grip strength, compared to the reference (25(OH)D 50 nmol/L), contractile force was −0.39 kg (CI −0.43, −0.35) lower at 25 nmol/l and −1.62 kg (CI −1.80, −1.44) lower at 10 nmol. In genetic analysis, each 10 nmol/L higher 25(OH)D was associated with 0.11 kg (95% CI 0.04, 0.19) greater contractile force (Figure 1B). Statistical evidence for non-linearity was observed using the residual method but not with the double-ranked method (corrected *p* = 5.28 × 10^−9^ and *p* = 0.27, respectively, Figure S7A,B) [23], with a visually similar association regardless of the approach.

### 3.2. Probable Sarcopenia

We observed a non-linear relationship between measured 25(OH)D and sarcopenia, with the simple and fully adjusted phenotypic models both following closely in shape (Figure 1C). The highest odds ratios (OR) for probable sarcopenia were observed <25 nmol/L of measured 25(OH)D. Compared to 50 nmol/L of measured 25(OH)D, the fully adjusted OR of probable sarcopenia was 9% higher for those at 25 nmol/L (OR 1.09, 95% CI 1.07, 1.12) and 52% higher at 10 nmol/L (OR 1.52, 95% CI 1.37, 1.69). In the genetic analysis (Figure 1D), there was some evidence for lower odds of probable sarcopenia by higher 25(OH)D (OR 0.96 (95% CI 0.92, 1.00). There was no evidence for non-linearity in the association between 25(OH)D and sarcopenia in the genetic analyses (corrected *p* = 0.06 with the residual method and *p* = 0.7 for the ranked method). Phenotypic analyses suggested that the association between 25(OH)D and sarcopenia is modified by obesity (*p*-value for interaction 1.368 × 10^−12^). In the genetic analysis, there was no evidence for lower odds by higher 25(OH)D for sarcopenic obesity (OR 0.97, 95% CI 0.93, 1.02), while analyses on probable sarcopenia not concurring with obesity (i.e., body fat <25% in males and <35% in females) retained the evidence seen in the overall probable sarcopenia assessment, with a stronger odds ratio (OR 0.92, 95% CI 0.86, 0.98) (Figure 1H).

### 3.3. Arm Skeletal Muscle Mass

As seen in the phenotypic analyses (Figure 1G), adjustment for covariates had a strong effect on the observed association between 25(OH)D and arm skeletal muscle mass, with muscle mass decreasing below 25 nmol/L 25(OH)D and appearing to plateau from 50 nmol/L onwards in the adjusted analysis. In genetic analysis (Figure 1H), evidence for a linear association was present (*p*-non-linearity ≥ 0.2 with both approaches), with each 10 nmol/L higher 25(OH)D associated with a 0.01 kg of weight (95% CI 0.003, 0.02) greater muscle mass.

### 3.4. Sensitivity Analyses

**Figure 2:**
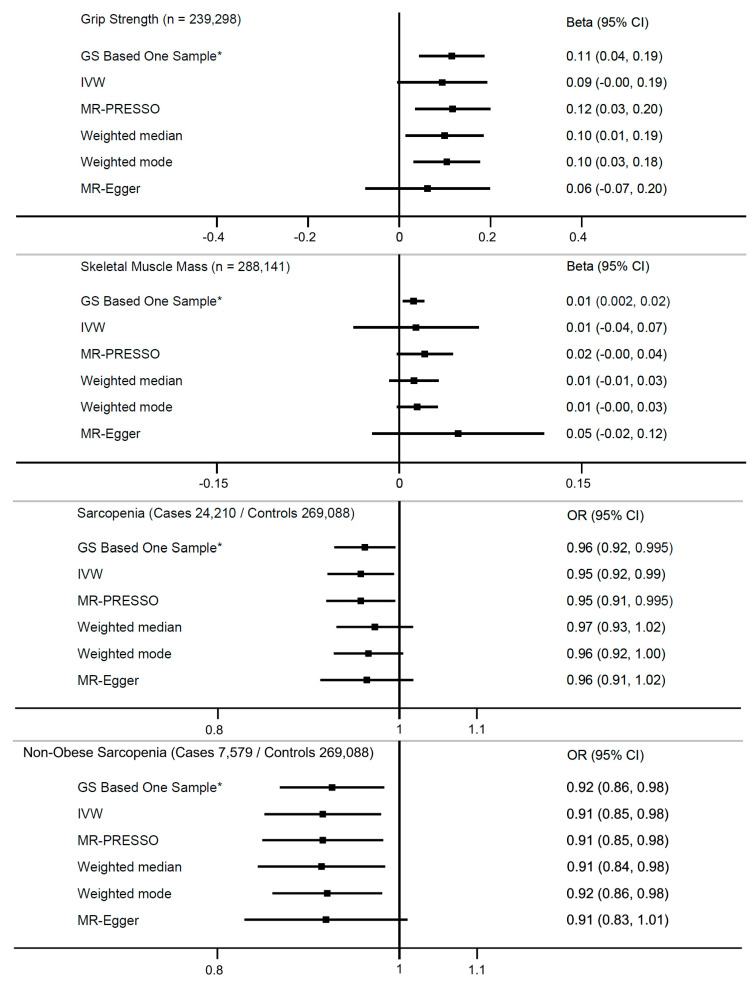
Mendelian randomization sensitivity analyses of genetically predicted 25(OH)D and grip strength, skeletal muscle mass, and probable sarcopenia in the UK biobank. * In the GS-based one-sample approach, the weight of SNPs are based on independent SNP-25(OH)D betta coefficients from the Sunlight Consortium [28]. Genetic analysis adjusted for age, sex, assessment centre, birth locations, SNP array, top 40 genetic principal components, and nuisance factors that could affect serum 25(OH)D measurements, including month in which blood sample was taken, fasting time before blood sample was taken, and sample aliquots for measurement. Outlier SNPs for MR PRESSO include the following: Grip Strength, rs1047891, rs212100, rs6782190; Skeletal Muscle Mass, rs1047891, rs35408430, rs6782190, rs7528419, rs76798800.

Linear analyses using different MR approaches supported the primary MR relationships (Figure 2). Similar results were obtained from sensitivity analyses produced using the 122 version of the vitamin D genetic score and when using the leave-block-out approach (Table S3) [23].

## 4. Discussion

Age-related decline of muscle strength and reduction in muscle composition are increasing concerns in ageing populations. In addition to an expected decrease of up to 30% of skeletal muscle mass by age 80 years [34], a subset of the general population is affected by above average sarcopenic conditions that can exacerbate a range of concurrent chronic health conditions, as well as increase the likelihood of suffering acute injuries [35]. The economic burden of muscle weakness has been a focus of research for several decades [36], with an example being the southern England-based Hertfordshire Cohort Study, where excess costs associated with muscle weakness—ascertained using sex-specific cut-offs for weak grip strength—were estimated to contribute GBP 2.5 billion to healthcare expenditure in the UK annually [37]. The increasing recognition of sarcopenia and muscle weakness as major contributors of disease burden has led researchers to call for a greater focus on the role and costs such challenges will have on healthcare systems moving forward into the future [38,39,40,41].

The considerable size of the UK Biobank has allowed our study to safely and ethically present evidence for a causal relationship between 25(OH)D concentration and suspected skeletal muscle pathophysiology in the form of probable sarcopenia and sarcopenic obesity, helping to inform this relatively underexplored area. Importantly, our study reaffirms the muscle trait MR associations (physiological—grip strength [15,16], and anatomical—skeletal muscle mass [18]), the mounting evidence of which collectively support the VDR knockout studies [8,9], which demonstrated skeletal muscle developmental issues, decreased lean mass, and reduced grip strength in affected mice.

The lack of supportive evidence in our study for the reducing of sarcopenic obesity risk by higher 25(OH)D was surprising, given the robust correlation seen in phenotypic research between vitamin D deficiency and both obesity- and non-obesity-related sarcopenia [7]. Our results—which show the evidence for probable sarcopenia remaining strongest in those without obesity—potentially suggest that vitamin D’s contribution to sarcopenia prevention/remediation is less able to overcome the conditions associated with obesity. Sarcopenic obesity has been associated with varying increases of inflammatory factors—specifically IL-6, C-reactive protein, IL-1 receptor antagonist, and soluble IL-6 receptor [42]—and while vitamin D has been shown to reduce pathological levels of inflammation (particularly interleukin 6 [43]), it could arise that resulting shifts in metabolic demand (resulting from obesity-related inflammation [44]) might overwhelm the influence vitamin D has on maintaining musculoskeletal homeostatic processes.

In the same way that emerging clinical trials (with non-obese sarcopenia sufferers) are demonstrating some consistent successes with the combining of vitamin D and branch chain amino acids [7], future clinical trials focusing on sarcopenic obesity could possibly consider co-administration of vitamin D with other anti-inflammatory nutraceutical or pharmaceutical agents. While obese individuals are known to already have an excess of branch chain amino acids (relative to healthy weight individuals [45]), further clinical trials for sarcopenic obesity could elucidate if co-administration of supplemental protein is appropriate in this group, and whether co-administration with additional anti-inflammatory factors support potential benefits from vitamin D in the presence of obesity and increased inflammation.

In addition to sarcopenia, there are other vitamin D-related aetiologies which may explain grip strength weakness, including adrenal dysregulation [46], metabolic syndrome [47,48], and anaemia [49,50]. For instance, the relation between 25(OH)D and serum ferritin [51] (and subsequently anaemia and grip strength [49,50]), as well as vitamin D and its role in endocrinology and metabolism more generally [52], may be areas via which the vitamin has indirect effects on skeletal muscle function and pathophysiology. Similarly, sarcopenia also predicts mortality, which itself is causally associated with 25(OH)D deficiency [53,54,55]. Further research focusing on broader mechanisms linking vitamin D and skeletal muscle, and their interactions with wider pathophysiology, is warranted.

Prior in vitro research and the VDR knockout studies provide evidence for active vitamin D having an effect on both skeletal muscle myocyte mass and function, with the latter likely being driven by myocyte mitochondrial regulation [56] and non-transcriptional membrane-associated signalling mechanisms that govern the contractile force of the muscle unit [57]. This dualistic effect of active vitamin D on skeletal muscle myocytes likely accounts for why we see a robust grip strength association in our study despite the modest anatomical variation in skeletal muscle mass by 25(OH)D concentrations. Although the evidence for a causal link between 25(OH)D and skeletal muscle mass observed in this study is notable, our study would suggest that vitamin D-mediated skeletal muscle mass stimulation is likely to be only one factor related to skeletal muscle development and maintenance, and improvements to mass in the healthy are minor and should not be interpreted as a reason to pursue excess of 25(OH)D sufficiency. More research is needed to establish whether ensuring sufficient vitamin D intakes may help to prevent age-related decline in muscle strength, and the extent to which related effects may depend upon the presence of excess adiposity.

### Strengths and Limitations

A primary strength of our study is the genetic approach, which has facilitated safe exploration of causal relationships pertaining to differences in 25(OH)D concentrations, including the exploration of associations in participants with deficiency, in whom it would otherwise be unethical to conduct properly controlled RCTs. Our study is possible because of 25(OH)D serum measurements and genotyping in 307,281 individuals by the UK Biobank, which provided sufficient power for analyses on both physiological and anatomical skeletal muscle outcomes. While we do not know if the probable sarcopenia measure used in this study is deterministic of a clinical diagnosis in the assessed population, the use of grip strength cut-offs is supported by the European Working Group on Sarcopenia in Older People as a clinical screening tool [20], and further research [37] has affirmed the public health relevance of these cut-offs. While future studies using more complex clustering of sarcopenia measures would be advantageous, our results remain highly pertinent given the emerging public health relevance being attributed to weak grip strength measures alone [58]. Although the grip strength cut-offs could reflect issues not directly related to skeletal muscle dysfunction, our study supports consideration of vitamin D deficiency as a determinant of grip strength, even in instances outside of clinically diagnosed sarcopenia.

We restricted the analyses to participants of white European descent, and while this reduces potential bias from population stratification, transferability to other ethnic groups may be limited. With a 5% response rate, the UK Biobank is not representative of the general UK population [59]. However, prior studies using the UK Biobank have replicated expected exposure–disease associations [59], with the MR approach shown to be less affected by selection bias than other types of observational studies, suggesting limited influence on our findings [60]. The MR method provides support for a causal association; however, estimates can be biased by horizontal pleiotropy [12]. Importantly, the instrument used for our analyses was constrained to variants with replicated evidence for an association with 25(OH)D [61]. Furthermore, our analyses include the use of several pleiotropy robust approaches, and there was no evidence for an association between the genetic score and potential confounders, either in the overall analyses or within stratums of residual serum 25(OH)D concentrations. As is the case with all MR analyses, genetic instruments approximate average effects over the life course. Therefore, it is possible that the true associations may be more complex than presented here, although as muscle degradation reflects a long-term process, there is an advantage to the MR approach in this respect.

## 5. Conclusions

Our study supports a causal relationship between 25(OH)D and skeletal muscle health. While evidence for the benefits did not extend to a lower risk of sarcopenic obesity, effective vitamin D-deficiency prevention strategies may help reduce age-related muscle weakness.

## Figures and Tables

**Table 1:** Demographic characteristics of UK Biobank participants.

		25(OH)D	25(OH)D <25 nmol/L	Grip Strength ^a^	Probable Sarcopenia	Sarcopenic Obesity	Arm Skeletal Muscle Mass ^b^
		*N* = 307,281	*N =* 36,009	*N =* 306,967 ^c^	*N =* 25,414	*N =* 16,520	*N =* 302,112 ^d^
	*N* (%)	Mean (SD)	%	Mean (SD)	%	%	Mean (SD)
All	307,281	49.82 (20.96)	11.70	31.04 (11.03)	8.28	5.54	5.51 (1.58)
Sex							
Men	144,538 (47.4)	49.86 (21.03)	11.60	39.61 (8.74)	6.39	3.98	6.92 (1.09)
Women	162,743 (53.0)	49.78 (20.89)	11.80	23.43 (6.22)	9.96	6.94	4.26 (0.60)
Age							
<60	169,594 (55.2)	48.38 (21.12)	13.46	32.48 (11.18)	5.46	3.40	5.56 (1.66)
≥60	137,687 (44.8)	51.57 (20.62)	9.54	29.28 (10.58)	11.75	8.23	5.44 (1.48)
BMI							
Low 25%, 12.1–24.0	76,633 (24.9)	53.04 (22.09)	10.19	28.62 (9.62)	7.61	1.21	4.53 (1.13)
Mid 50%, 24.1–29.8	153,312 (50.0)	50.95 (20.68)	10.05	32.19 (11.17)	7.55	5.46	5.57 (1.44)
High 25% 29.8–74.7	76,667 (25.0)	44.42 (19.23)	16.40	31.23 (11.63)	10.18	9.77	6.36 (1.71)
Missing	669 (0.2)	40.69 (21.11)	26.01	25.51 (12.86)	33.73	4.33	5.34 (1.48)
Location ^e^							
South, ≤51° Lat	102,226 (33.3)	51.43 (20.49)	9.29	30.77 (10.84)	8.35	5.20	5.53 (1.58)
Mid, 52–53° Lat	144,470 (47.0)	49.93 (20.88)	11.35	31.10 (11.11)	8.42	5.80	5.51 (1.58)
North, 54–≥55° Lat	60,585 (19.7)	46.82 (21.59)	16.62	31.36 (11.16)	7.83	2.49	5.45 (1.58)
Smoking							
Non-smokers	167,537 (54.5)	50.04 (20.63)	10.92	30.48 (11.00)	8.07	5.22	5.36 (1.55)
Ex-smokers	108,015 (35.2)	50.77 (21.04)	10.66	31.61 (10.99)	8.37	6.05	5.69 (1.60)
Current smokers	30,673 (10.0)	45.22 (21.80)	19.63	32.23 (11.17)	8.91	5.31	5.66 (1.60)
Missing	1056 (0.3)	50.17 (21.77)	12.41	28.95 (11.14)	14.58	11.22	5.57 (1.60)
Alcohol							
Daily	65,476 (21.3)	51.22 (21.51)	11.03	33.09 (10.82)	6.51	3.93	5.71 (1.56)
1 to 4 times wk	155,474 (50.6)	50.87 (20.80)	10.19	31.85 (11.01)	7.11	4.67	5.58 (1.60)
1 to 3 times mo	34,061 (11.1)	48.08 (20.26)	12.91	29.56 (10.73)	8.46	5.85	5.35 (1.58)
Special occasion	32,125 (10.5)	46.18 (20.40)	15.74	27.05 (10.43)	12.85	9.37	5.13 (1.48)
Never	19,934 (6.5)	45.91 (20.95)	17.06	27.07 (10.66)	15.47	11.11	5.19 (1.45)
Missing	211 (0.07)	44.97 (21.05)	16.59	28.54 (11.32)	17.54	13.86	5.39 (1.57)
Physical activity							
Low	91,911 (29.9)	46.30 (20.20)	15.18	29.78 (10.96)	10.39	7.49	5.47 (1.59)
Moderate	149,064 (48.5)	50.60 (20.80)	10.50	31.31 (10.88)	7.17	4.65	5.47 (1.57)
High	59,518 (19.4)	54.01 (21.41)	8.11	32.65 (11.09)	6.37	3.78	5.62 (1.60)
Missing	6788 (2.2)	43.33 (21.32)	22.48	28.32 (12.19)	20.78	14.80	5.77 (1.70)
Education							
None	52,119 (17.0)	50.40 (21.43)	11.89	28.56 (10.98)	14.47	10.74	5.43 (1.55)
NVQ/CSE/A-Lev.	109,007 (35.5)	50.55 (21.19)	11.22	31.02 (11.18)	7.90	5.33	5.51 (1.61)
Deg./professional	143,586 (46.7)	49.04 (20.57)	12.00	31.99 (10.79)	6.23	3.79	5.54 (1.57)
Missing	2569 (0.84)	50.33 (20.90)	11.60	29.48 (11.22)	13.36	9.04	5.52 (1.59)
Townsend index							
Q1 Deprivation low	76,746 (25.0)	51.91 (20.71)	9.16	31.71 (11.06)	6.60	4.21	5.50 (1.57)
Q2	76,745 (25.0)	51.50 (20.70)	9.35	31.31 (11.10)	7.37	4.82	5.49 (1.58)
Q3	76,719 (25.0)	49.91 (20.79)	11.20	30.92 (10.98)	8.25	5.55	5.50 (1.59)
Q4 Deprivation high	76,711 (25.0)	45.94 (21.08)	17.11	30.24 (10.93)	10.90	7.62	5.54 (1.59)
Missing	360 (0.1)	50.02 (20.53)	11.39	31.37 (11.16)	7.22	4.84	5.61 (1.66)

Kg, kilograms; BMI, body mass index; NVQ, National Vocational Qualification; CSE, Certificate of Secondary Education; A-levels, Advanced levels; Q, quartiles; SD, standard deviation. ^a^ Average grip strength, min 0 kg to max 85 kg of contractile force, ^b^ Bioimpedance analysis-derived skeletal muscle mass of the combined arm, min 2 kg to 35.9 kg in weight, ^c^ Missing *n*= 314 (0.10%), ^d^ Missing *n* = 5169 (1.68%). ^e^ Location categorization derived from assessment centre aggregation by longitudinal zoning, as described in the Supplementary Materials (p. 4). *p*-values for all models <0.01; all models were adjusted for sex, age, assessment centre, and nuisance factors that could affect serum 25(OH)D measurements, including the month in which blood sample was taken, fasting time before blood sample was taken, and sample aliquots for measurement.

## Data Availability

Protocol: not available. Data: This research was conducted using the UK Biobank resource under application number 20175. An analytic code will be made available upon request, and all data and code will be available to approved users upon application to the UK Biobank.

## Supplementary Materials

The following supporting information can be downloaded at: https://www.mdpi.com/article/10.3390/nu15122703/s1, Figure S1: Study population and participant inclusion criteria; Figure S2: Mendelian randomization assumption diagram; Figure S3: Schematic representation of Mendelian randomization analyses performed in the current study; Figure S4: Distribution of the vitamin D genetic score (mapped on an X-axis by increasing number of alleles) in the UK Biobank (A), and the association of the vitamin D genetic score (in six groups—lowest to highest number of alleles) with measured 25(OH)D concentrations in the UK Biobank (B); Figure S5:Association of the vitamin D genetic score with potential confounders across 100 strata of residuals of measured 25(OH)D; Figure S6: Mean kilogram difference in arm skeletal muscle mass by probable sarcopenia status in UK Biobank participants; Figure S7: Residual method (A) and doubly rank method (B) non-linear Mendelian randomization analysis of genetically predicted 25(OH)D on grip strength, projected on the measured 25(OH)D scale; Figure S8: Selection of variants for the genetic instrument for measured 25(OH)D concentrations; Figure S9: Distribution of the GRS-measured 25(OH)D association across 100 strata of residual measured 25(OH)D; Table S1: Association of the vitamin D genetic score with potential confounders in the UK Biobank; Table S2: Functional blocks used in the leave-block-out analyses; Table S3: 122 SNP genetic score and leave block out analysis; Table S4: Demographic characteristics of UK Biobank participants—including height and waist circumference; Table S5. Genome-wide significant vitamin D variants used for the genetic instruments for measured 25(OH)D concentrations. References [25,26,28,30,31,32,62,63,64,65,66,67,68,69,70,71,72,73] are cited in supplementary file.
