# Supplementary material for: Muscle Traits, Sarcopenia, and Sarcopenic Obesity: A Vitamin D Mendelian Randomization Study
Source: Nutrients. 2023 Jun 9;15(12):2703. doi: 10.3390/nu15122703 (PMC10301137; doi:10.3390/nu15122703)
Supplement: Supplementary file 1 [file nutrients-15-02703-s001.zip › nutrients-2436523-supplementary.pdf]

Muscle traits, sarcopenia, and sarcopenic obesity: a vitamin D Mendelian randomization study – Supplementary Appendix.

Table of Contents:

Measurement of vitamin D status	**03**
Covariates	**03**
Genetic instruments using 35 variants	**05**
Genetic instrument using 122 vitamin D GWAS variants	**05**
Linear Mendelian randomization	**06**
Leave-block-out analysis	**08**
Non-linear Mendelian randomization	**08**
**Figure S1**. Study population and participant inclusion criteria.	**10**
**Figure S2**. Mendelian randomization assumption diagram.	**11**
**Figure S3.** Schematic representation of Mendelian randomization analyses performed in the current study.	**12**
**Figure S4.** Distribution of the vitamin D genetic score (mapped on an X-axis by increasing number of alleles) in the UK Biobank (A), and the association of the vitamin D genetic score (in six groups – lowest to highest number of alleles) with measured 25(OH)D concentrations in the UK Biobank (B).	**13**
**Figure S5.** Association of the vitamin D genetic score with potential confounders across 100 strata of residuals of measured 25(OH)D.	**14**
**Figure S6.** Mean kilogram difference in arm skeletal muscle mass by probable sarcopenia status in UK Biobank participants.	**15**
**Figure S7.** Residual method (A) and doubly rank method (B) non-linear Mendelian randomization analysis of genetically predicted 25(OH)D on grip strength, projected on the measured 25(OH)D scale.	**16**
**Figure S8.** Selection of variants for the genetic instrument for measured 25(OH)D concentrations.	**17**
**Figure S9.** Distribution of the GRS-measured 25(OH)D association across 100 strata of residual measured 25(OH)D.	**18**
**Table S1.** Association of the vitamin D genetic score with potential confounders in the UK Biobank.	**19**
**Table S2.** Functional blocks used in the leave-block-out analyses.	**20**
**Table S3.** 122 SNP genetic score and leave block out analysis.	**22**
**Table S4.** Demographic characteristics of UK Biobank participants – including height and waist circumference.	**23**
**Table S5.** Genome-wide significant vitamin D variants used for the genetic instruments for measured 25(OH)D concentrations.	**24**
**References**	**28**

Authors:

Joshua P Sutherland[1], Ang Zhou[1,2], Elina Hyppönen[1,2]

[1]Australian Centre for Precision Health, Clinical and Health Sciences, University of South Australia, Adelaide, Australia.

[2]South Australian Health and Medical Research Institute, Adelaide, Australia

Corresponding author: Prof Elina Hyppönen; Australian Centre for Precision Health, Clinical and Health Sciences, University of South Australia, Adelaide, Australia, GPO Box 2471, Adelaide SA 5001; +61 8 830 22518; Elina.Hypponen@unisa.edu.au

Supplementary Methods:

Measurement of vitamin D status

The LIAISON XL 25(OH)D assay (DiaSorin, Stillwater, USA) is a magnetic microparticle separation technology, fully automated, chemiluminescence immunoassay device.[1] Baseline serum 25(OH)D concentrations were measured from blood samples with a mean fasting time of 3.8 hours (SD 2.4). Assay precision (CLSI EP5-A2), produced a coefficient of variation (CV) of 2.3% (intra-run) and a CV 7.8% (total), conducted using 6 samples and 2 controls.[1] The LIAISON XL has a functional sensitivity of ≤=4.0 ng/ml, in accordance with Clinical Laboratory and Standards Institute (CLSI) EP17-A protocols.[1] 25(OH)D reflect total concentrations, including both 25(OH)D_2 and 25(OH)D_3.

Covariates

As described in the primary manuscript, covariates used in adjustments were acquired at baseline – at the assessment centre most closely located to a respective participant's residential location – from self-reported, touchscreen questionaries (age, sex, physical activity, smoking, alcohol, birth location), residential address data (Townsend deprivation index)[2] physical assessments (height and waist circumference). The assessment centre at which baseline assessment occurred was itself used as a covariate, constructed as per the following: Leeds (n= 28,105), Bristol (n=27,983), Newcastle (n=23,755), Nottingham (n=21,864), Liverpool (n=20,438), Sheffield (n=19,352), Reading (n=19,297), Hounslow (n=13,377), Bury (n=18,690), Croydon (n=13,556), Birmingham (n=14,252), Middlesborough (n=13,806), Stoke (n=12,785), Glasgow (n=11,697), Cardiff (n=11,959), Edinburgh (n=11,327), Oxford (n=9,348), Manchester (n=8,524), Barts (n=5,257), Swansea (n=1,449), and Wrexham (n=460). Height data was acquired from a Seca 202 stadiometer. For Townsend deprivation index, each participant was assigned a score corresponding to socioeconomic data attributed to their postcode; this was derived from the preceding UK census of population and housing.[2,3] 25(OH)D and the remaining genetic related covariates were derived from baseline biological sample acquisition data (month in which blood sample was taken, fasting time before blood sample was taken, and top 40 genetic principal components) and

subsequent laboratory data [sample aliquots for 25(OH)D measurement and Single Nucleotide Polymorphism (SNP) array data]. Finally, two variables were used exclusively for presenting demographic characteristic purposes (**main text Table 1 and Table S4**), these being longitudinal location and BMI. Due to the relevance of UVB exposure in vitamin D analyses, assessment centre information was presented as longitudinal location categories: <=51° (Croydon, Barts, Hounslow, Reading, Oxford, Bristol, Cardiff, Swansea), 52°-53° (Birmingham, Stoke, Nottingham, Wrexham, Liverpool, Bury, Manchester, Sheffield, Leeds), and 54°-≥55° (Middlesborough, Newcastle, Glasgow, Edinburgh). Body mass index (BMI) was derived from height and weight measures collected at baseline (calculated as body weight [kilograms] divided by height squared [meters]).[4] Height and waist circumference were chosen for phenotypic adjustments – to account for body shape and obesity – rather than using body mass index (BMI), due to BMI's calculation explicitly including measure of skeletal muscle mass via body weight; BMI was nonetheless used for descriptive purposes, due its common use and familiarity. All phenotypic analyses were restricted to the sample with full information on covariates included in the full model (skeletal muscle mass: observations n = 291,847; grip strength: observations n = 296,013; probable sarcopenia controls n = 272,555 / cases n = 23,458); and sarcopenic non-obesity controls n = 272,555 / cases n = 7,580. For the genetic analyses, we included sex, age, vitamin D month, assessment location, birth location, principal components, fasting, vitamin D aliquot, and SNP array (skeletal muscle mass: observations n = 288,697; grip strength observations n = 293,298; probable sarcopenia controls n = 269,088 / cases n = 24,210; and sarcopenic non-obesity controls n = 269,088 / cases n = 7,579). (**Figure S1**).

Genetic instruments using 35 variants

We constructed a weighted genetic score wherein common autosomal SNPs (i.e., minor allele frequency >5%) were collated from a genome-wide association analysis (GWAS) for measured 25(OH)D serum concentration in UK Biobank; 143 independent loci were identified.[5] Subsequently, we replicated our SNP analyses in the previous SUNLIGHT consortium GWAS,[6] and this left 35 SNPs that showed a consistent direction and a p value of <0.05 (**Figure S8**). The process of restricting our instrument to replicated SNPs has enabled us to ensure the observed GWAS signals are robust. Additionally, we were able to use the SUNLIGHT consortium meta-analyses derived effect estimates [for the SNP association with measured 25(OH)D] as the individual SNP weights; this allowed us to avoid bias due to sample overlap.[7] For our vitamin D genetic score, the independently derived weighted average of the number of measured 25(OH)D-increasing alleles for an individual were computed, and then multiplied by the number of available variants.

Genetic instrument using 122 vitamin D GWAS variants

We constructed an alternative genetic instrument for sensitivity analyses, in which a broader set of variants (122 autosomal single nucleotide polymorphisms, SNPs) that associated with measured 25(OH)D concentrations were included (Figure S8). As these 122 SNPs were discovered in the UK Biobank,[5] we used the 10-fold cross-validation approach[7] to construct the 122 SNP version of the vitamin D genetic score; this was done to mitigate any potential bias caused by using the internal weights. This process involved the random division of samples into 10 equal sub-samples, with weights for each sub-sample taken from the other 9 sub-samples. Information for the 122 SNPs can be found in **Table S5**.

<u>Linear Mendelian randomization:</u>

In the primary Genetic Score One Sample method, linear MR estimate is computed using the ratio of coefficients method[8] where the vitamin D genetic score – measured 25(OH)D and the vitamin D genetic score – outcome association estimates are computed and then used to calculate the ratio estimator. The vitamin D genetic score-25(OH)D and vitamin D genetic score-outcome associations included adjustment for: age, sex, assessment centre, birth locations, SNP array, top 40 genetic principal components, and nuisance factors which could affect measured 25(OH)D, including month in which blood sample was taken, fasting time before blood sample was taken, and sample aliquots for measurement.

In order to address potential horizontal pleiotropy, we also use five SNP-based two-sample methods, including inverse-variance weighted method (IVW), Mendelian randomization Egger method, weighted median Mendelian randomization method, weighted mode method, and Mendelian randomization pleiotropy residual sum and outlier (MR Presso) test. These five methods are complementary to one another, in that each one is robust to different patterns of horizontal pleiotropy; to this end, a good agreement across the methods is suggestive of robust evidence of causal association. In contrast to the above outlined genetic score-based analysis, the SNP-based two-sample methods use SNP-level information and takes SNP-exposure and SNP-outcome association estimates as inputs (**Figure S3**). In our two-sample MR analyses, SNP-25(OH)D and SNP–outcome association estimates were all taken from UK Biobank. For grip strength and skeletal muscle mass, we applied the split-sample strategy to avoid bias due to overlapping samples.[9] More specifically, the full sample is randomly split into two subsamples of equal size (Samples A and B); the MR estimate is calculated for each sample (MR_A and MR_B) and then combined to obtain the overall estimate ($MR_{combined}$). MR_A is computed using SNP–outcome association estimates from Sample A and SNP–exposure association estimates from Sample B, whereas SNP-outcome association estimates from Sample B and SNP–exposure association estimates from Sample A are used to compute MR_B. MR_A and MR_B are then combined in a fixed-effects meta-analysis to compute $MR_{combined}$. For the sarcopenia analyses, we implemented a different strategy to avoid potential bias due to overlapping samples,[9] where we computed SNP-Probable sarcopenia association

estimates in the full sample but took SNP-25(OH)D association estimates from controls only. All five two-sample MR methods were conducted using the 35 vitamin D variants that were used to construct the vitamin D genetic score (Table S5). In IVW analysis, it is assumed that there is no pleiotropy of variants, or the net pleiotropy is zero. The estimator is obtained by the weighted regression of the effect estimates of variant-outcome association on the effect estimates of variant-exposure association,[10] with the weight being the inverse variance of variant's ratio estimate. Like the IVW method, Mendelian randomization Egger also expects that the regression model additionally included an intercept term, which represents the average pleiotropic effect.[11] Conversely, the Mendelian randomization Egger method can account for directional pleiotropy, however, additional untestable assumptions are introduced, which state that pleiotropic effect must be independent of variant-exposure association. In Mendelian randomization Presso,[12] the IVW method is performed iteratively, wherein each variant is, in turn, omitted from the analysis. The residual sum of squares (RSS) is calculated at each iteration and used as a heterogeneity measure for the ratio estimate. The corresponding variant is considered as an outlier, if the observed RSS is substantially large in the null distribution of the expected RSSs. The causal estimate in MR Presso is obtained by the IVW method with all outlying variants removed. In the weighted mode method, variants (which are weighted by the inverse variance of their ratio estimates) are clustered into groups by the similarity of their ratio estimates.[13] The clustering is performed using a normal kernel density function, with its spread depending on a bandwidth parameter. The causal effect is estimated based on the cluster with the largest weight. An unbiased causal effect will be returned in the weighted mode method if the variants within the cluster with the largest weight are valid instruments. In the weighted median-based approach,[14] variants are ranked by their ratio estimates, with the causal estimate being the median of the distribution. The weighted median method will return a consistent estimate if at least 50% of the weight comes from valid instruments – median is taken from a distribution of the ratio estimates in which variants with more precise ratio estimates receive more weight.

Leave-block-out analysis

In our leave-block-out analysis, variants were grouped into functional blocks based on the traits that they are associated with from the PhenoScanner search. For sensitivity purposes, we then removed one block of variants at a time and repeated the non-linear Mendelian randomization analysis with the vitamin D genetic score that was constructed using the non-removed, remaining variants. This process was iterated through all functional blocks.

PhenoScanner V2 is a publicly available database that has been curated from the results of large-scale genetic association studies.[15] On 09/09/2021, we searched for evidence of variant-disease/trait associations in the PhenoScanner V2 database for all 35 variants. At the GWAS threshold (p<5E-08), 20 variants were associated with traits besides serum 25(OH)D (**Table S2**). We grouped variants into 4 functional blocks, based on their associated traits, including blocks for blood, lipids/metabolic, renal traits – and for variants whose associated traits did not fall into one of these 3 blocks, they were grouped together as the 'unclassified' block (Table S2-3).

Non-linear Mendelian randomization:

We examined non-linearity of genetic association using two available methods, including the residual method[16] and doubly-ranked method.[17] Both approaches involve stratifying the sample into 100 strata, but employ different stratification strategies. For the residual method, [16] strata are formed using 100 quantiles of residual measured 25(OH)D, which is defined as measured 25(OH)D minus the variation induced by the genetic score (correlation between residual measured 25(OH)D and measured 25(OH)D, *r*= 0.986). For the doubly ranked method, [17] strata are formed by first ranking participants into pre-strata based on their level of genetic score and then by ranking participants within each pre-stratum according to their

level of measured 25(OH)D. In both non-linear MR methods, stratum-specific MR estimate is computed using the ratio of coefficients method (as described in the linear MR analysis, see section linear MR), and then meta-regressed on the mean stratum measured 25(OH)D concentration. The meta-regression was performed by fitting a range of fractional polynomial exposure-outcome models of degree 1 and degree 2, with the best-fitting model being selected based on the likelihood ratio test. Conventionally, if the best-fitting fractional polynomial model of degree 1 has a better fit than the linear model[16] (i.e. $P_{Non\text{-}linear} < 0.05$), then a non-linear effect is evident – however, as per our use of the Bonferroni correction (outlined page 9 of main text) we used a stricter threshold for our analyses. In residual method non-linear MR analysis it is assumed that the association of genetic instrument with the exposure is constant over the entire distribution of exposure [here, measured 25(OH)D], and to test this assumption we computed the estimate for the association of the vitamin D genetic score and measured 25(OH)D in each of 100 strata and then examined the heterogeneity between strata using the trend test and Cochran Q test.[16] As we observed an apparent violation in the constant genetic effect assumption arising from 1st and 100th strata (**Figure S9**), when performing the non-linear analysis using the residual method, we excluded these two outlying strata to minimize the impact of any potential violation in the constant genetic effect assumption.

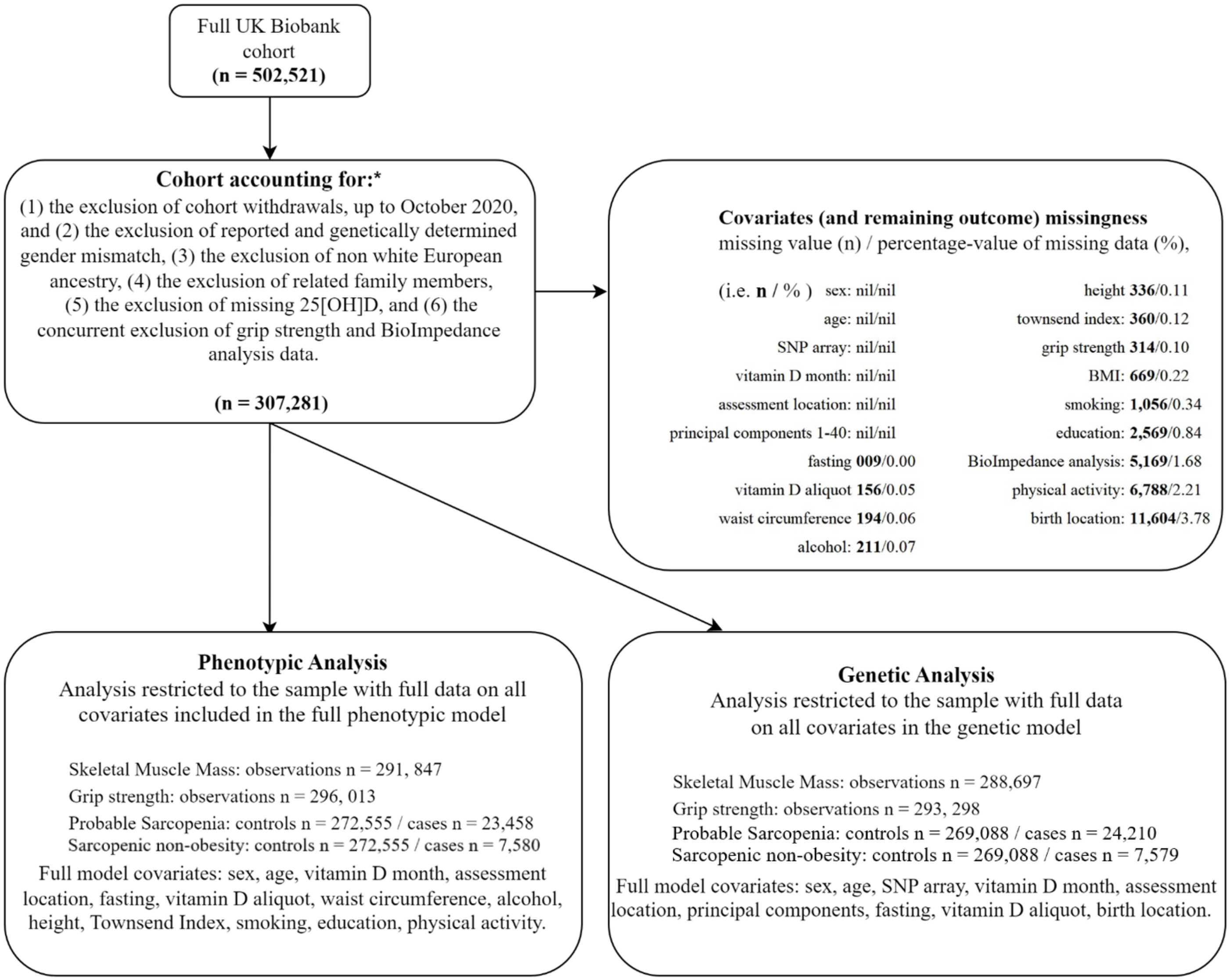

Figure S1. Study population and participant inclusion criteria *Exclusion was done in a sequential order.

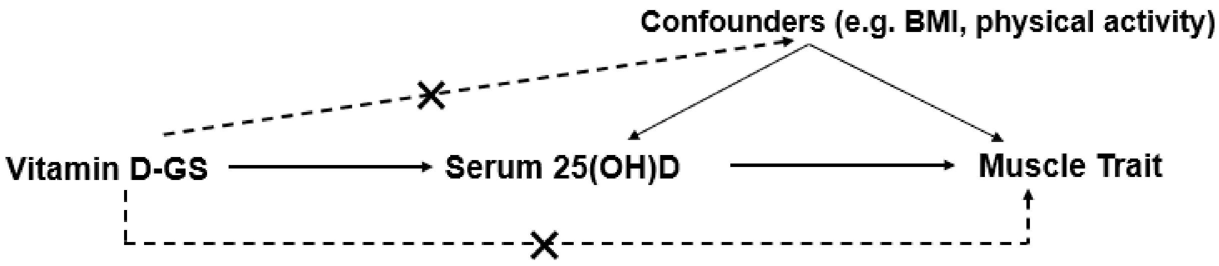

Figure S2. Mendelian randomization assumption diagram.

Valid causal inference from the Mendelian randomization analysis relies on 3 key assumptions:[18] 1) vitamin D genetic score associates with measured 25(OH)D concentrations; 2) vitamin D genetic score has no direct effect on outcome; 3) vitamin D genetic score does not associate with confounders of measured 25(OH)D and outcome. Vitamin D-GS: vitamin D genetic score.

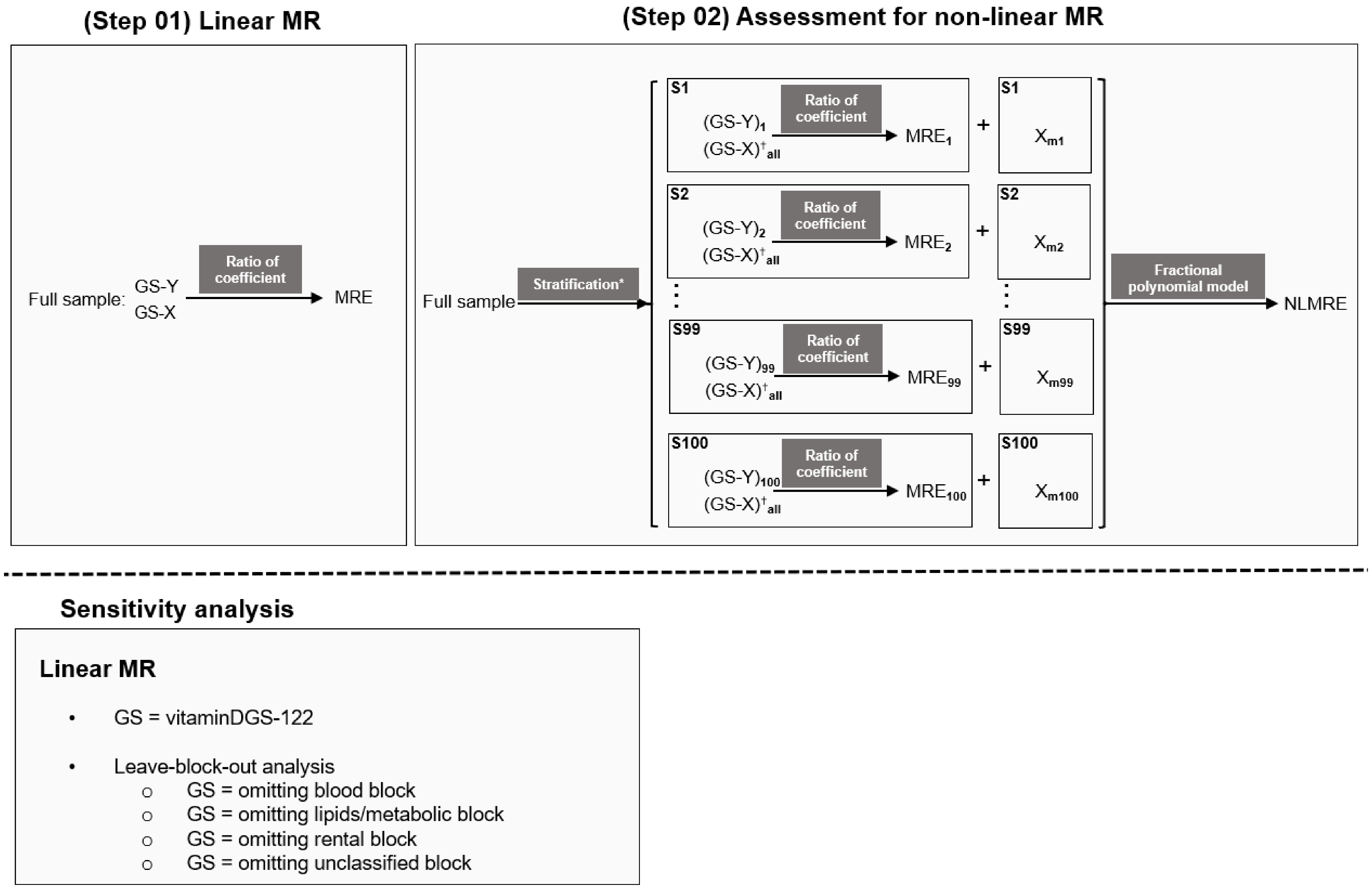

Figure S3. Schematic representation of Mendelian randomization analyses performed in the current study.

MR: Mendelian randomization; GS: genetic score; SNP: single nucleotide polymorphism; S_n: the nth stratum; MRE: MR estimates; NLMRE: non-linear MR estimates; GS-X: beta of GS-exposure association; GS-Y: beta of GS-outcome association; SNP-X: beta of SNP-exposure association; SNP-Y: beta of SNP-outcome association; *The residual method and doubly ranked method use different stratification methods. For the residual method, strata were formed using quartiles of residual measured 25(OH)D. For the doubly ranked method, strata were formed by first ranking participants into pre-strata based on their level of genetic score and then by ranking participants within each pre-stratum according to their level of measured 25(OH)D. Stratification using residual measured 25(OH)D; In non-linear MR: $(GS\text{-}X)^{\dagger}_{all}$: for the residual method GS-X is taken from the full sample, while for the doubly ranked method, GS-X is taken from the corresponding stratum; $(GS\text{-}Y)_n$: GS-Y from the nth stratum; X_{mn}: median of exposure in the nth stratum; In secondary analysis, stratified MR: S1: < 25 nmol/L; S2: 25-49.9 nmol/L; S3: 50-74.9 nmol/L; S4: >=75 nmol/L. In sensitivity analysis, stratified MR: $S1^{\$}$: < 25 nmol/L; $S2^{\$}$: >=25 nmol/L; S3: 50-74.9 nmol/L; S4: >=75 nmol/L $(SNP\text{-}Y)_n$: SNP-Y from the nth stratum; $(SNP\text{-}X)_{control}$: for sarcopenia analyses, SNP-X from non sarcopenia case individuals in the full sample; †Two-sample methods include Inverse Variance Weighted MR, MR-Egger, Weighted median MR, Weighted mode MR, and MR PRESSO.

A

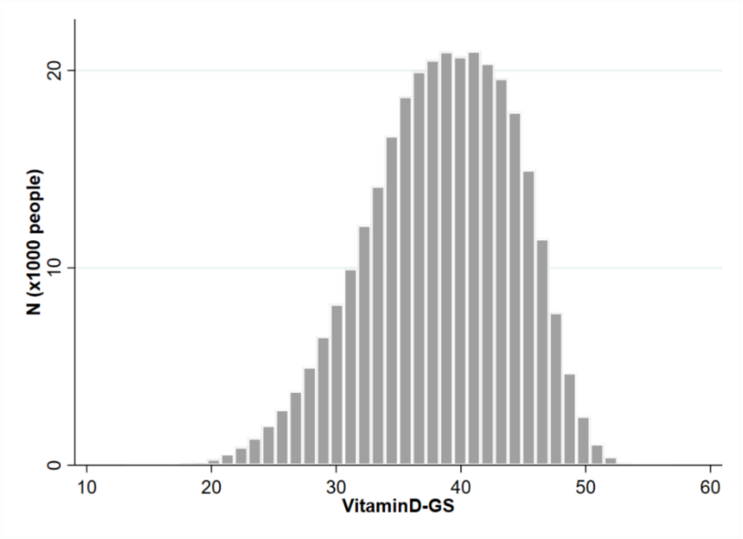

B

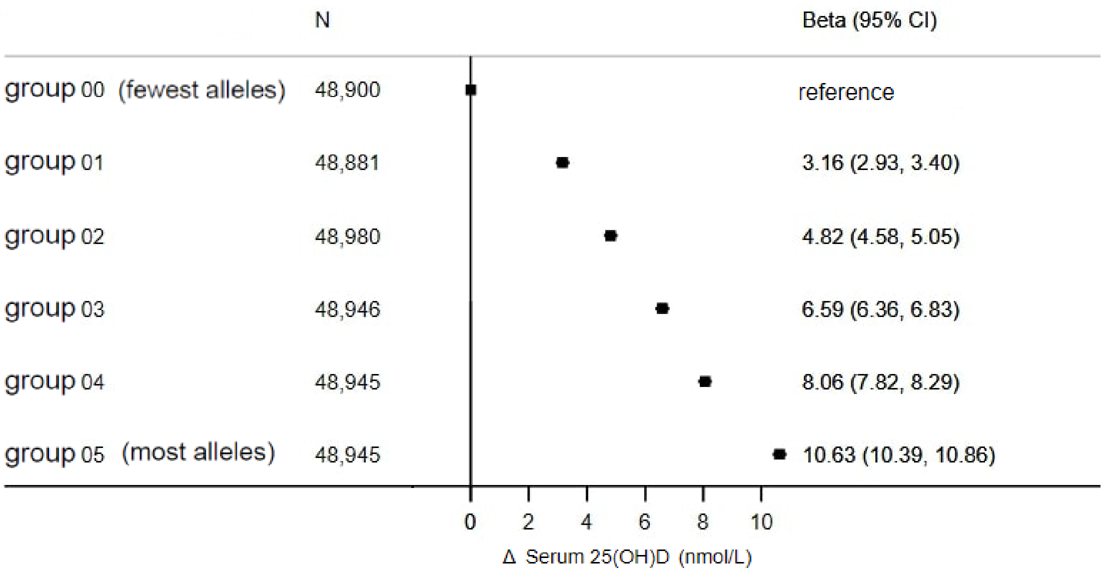

Figure S4. Distribution of the vitamin D genetic score (mapped on an X-axis by increasing number of alleles) in the UK Biobank (A), and the association of the vitamin D genetic score (in six groups – lowest to highest number of alleles) with measured 25(OH)D concentrations in the UK Biobank (B).

The association was tested using the linear regression, with the model adjusted for age, sex, genotyping array, birth location, assessment center location, top 40 genetic principal components, and nuisance factors which could affect serum 25(OH)D measurements, including month in which blood sample was taken, fasting time before blood sample was taken, and sample aliquots for measurement. Error bars are 95% confidence intervals.

Vitamin D-GS: Vitamin D Genetic Score

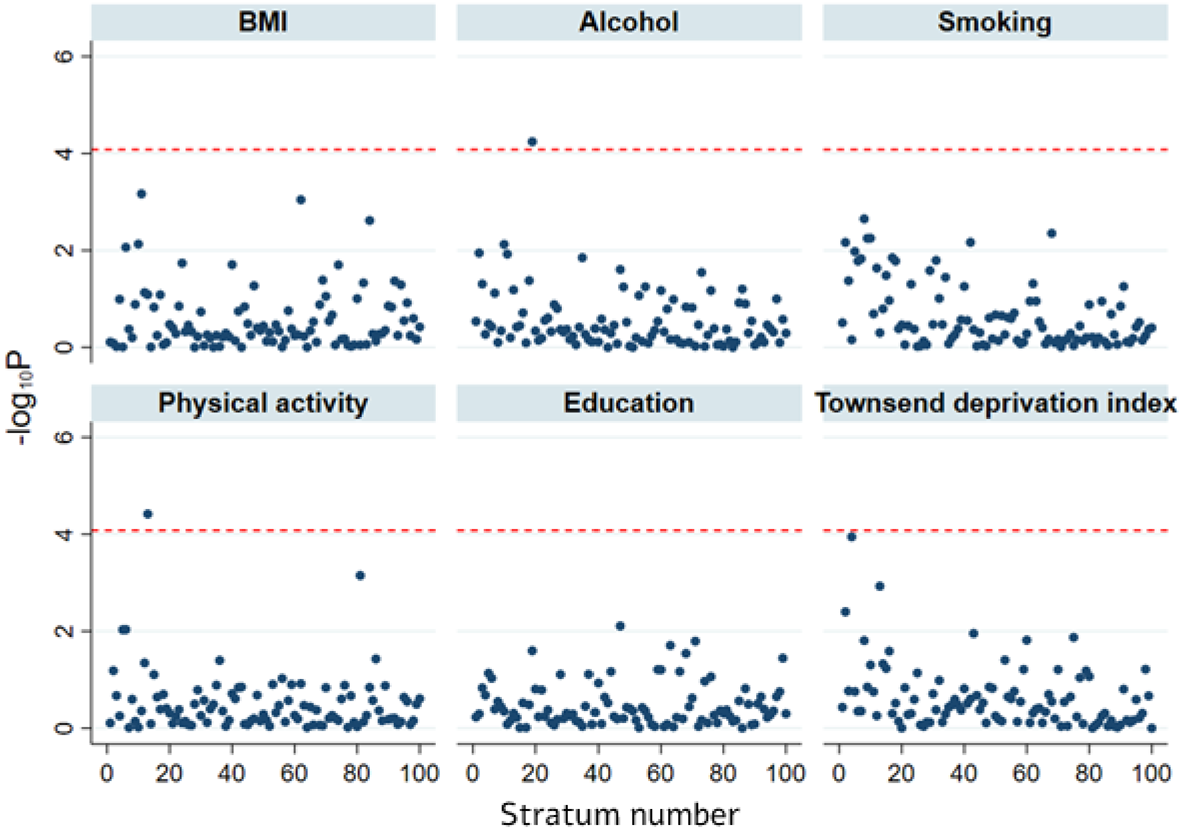

Figure S5. Association of the vitamin D genetic score with potential confounders across 100 strata of residual measured 25(OH)D.

Potential confounders include BMI, alcohol intake, smoking status, physical activity level, education attainment and Townsend deprivation index. $P_{threshold}$ = 0.05/(6 confounders x 100 strata) = 8.3E-5. Dashed line represents -$\log_{10}$P at $P_{threshold}$.

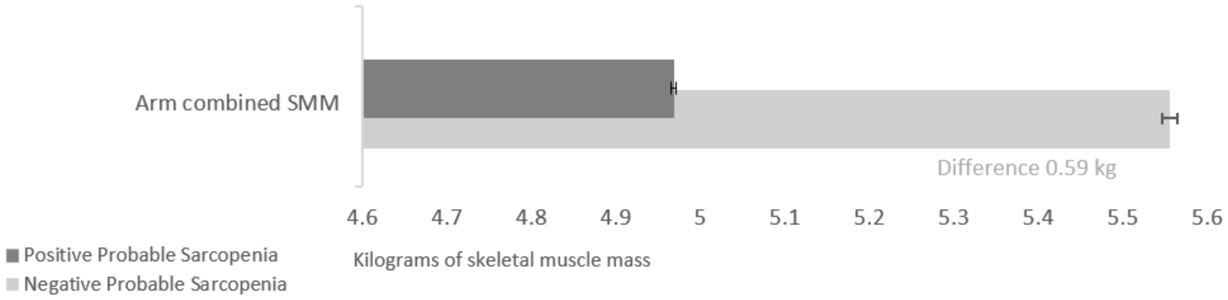

Figure S6. Mean kilogram difference in arm skeletal muscle mass by probable sarcopenia status in UK Biobank participants.
SMM: Skeletal Muscle Mass

**A. Grip Strength - Residual Method**	**B. Grip Strength – Doubly Rank Method**
Corrected $P_{Non\text{-}linearity}$ 5.28 x 10-9 (Corrected $P_{Linear\text{-}trend}$ 0.006)	$P_{Non\text{-}linearity}$ 0.27* (Corrected $P_{Linear\text{-}trend}$ 0.006)

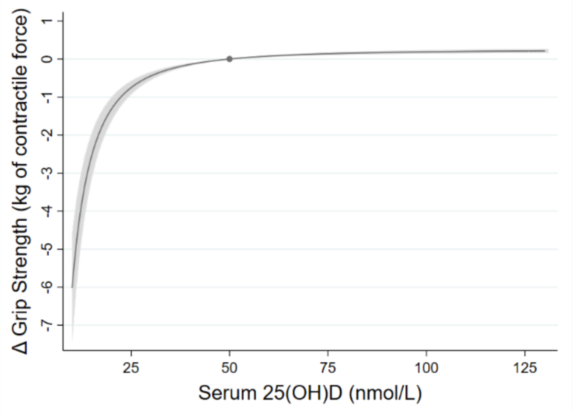

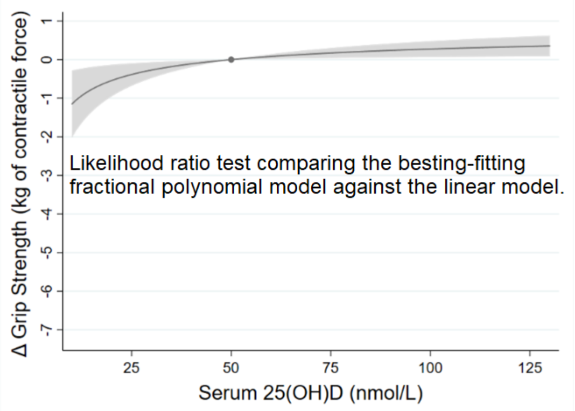

Figure S7. Residual method (A) and doubly rank method (B) non-linear Mendelian randomization analysis of genetically predicted 25(OH)D on grip strength, projected on the measured 25(OH)D scale.

In both methods the sample is stratified into 100 strata. For the residual method we removed the 1st and 100th strata because an apparent violation in the constant genetic effect assumption, as per Figure S8. Refer to Figure 1, main text, for full accompanying information on genetic analysis model adjustments and figure elements.

*The doubly ranked method, it may have a limited statistical power to detect an effect at low 25(OH)D concentration. We estimated statistical power (with respect to grip strength) across 25(OH)D distribution by calculating stratum-specific power of detecting a linear genetic effect across 4 strata of 25(OH)D. Statistical power (PMID: 24608958) of detecting a 0.022 SD change in grip strength by SD increase in 25(OH)D from the lowest to the highest stratum was 18.1%, 29.3%, 39.9% and 40.2%, respectively. The estimate for the association for which the power was calculated was taken from the linear MR analysis and re-scaled to reflect SD change in grip strength per SD increase in 25(OH)D (SD for 25(OH)D = 20.96 nmol/L, SD for grip strength = 11.03 kilograms of contractile force). The differences in statistical power across strata are driven by differences in instrument strength (R2), which are 0.031, 0.057, 0.083, and 0.083 from the lowest to the highest stratum, respectively.

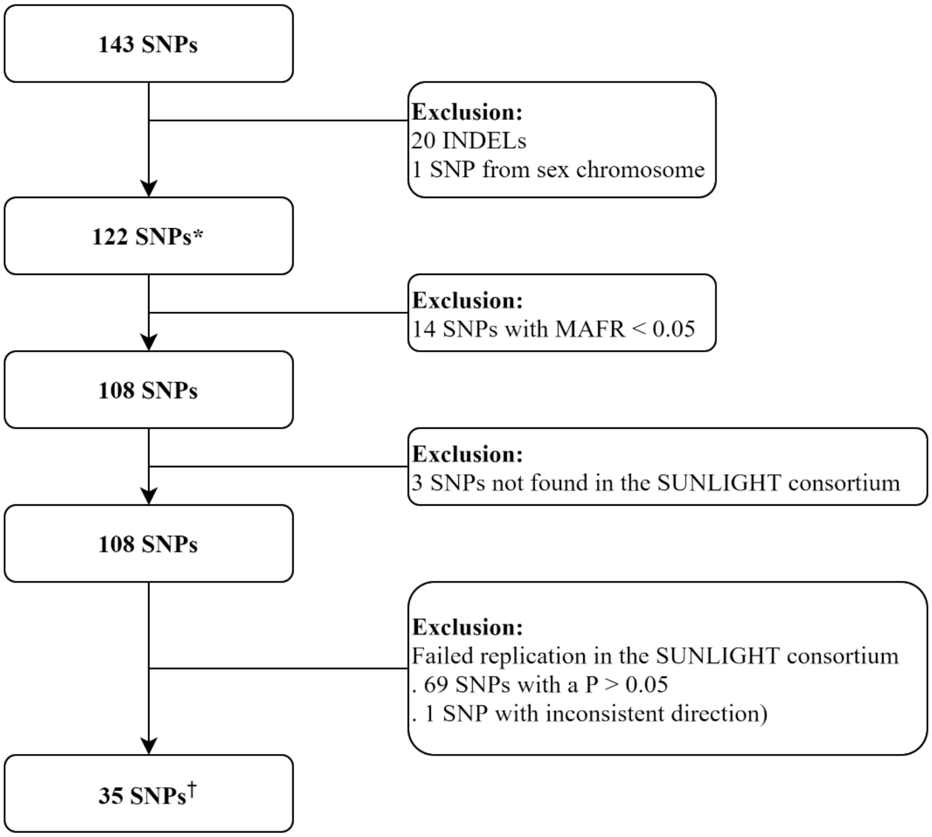

Figure S8. Selection of variants for the genetic instrument for measured 25(OH)D concentrations.

SNP: single nucleotide polymorphism. *SNPs used for the vitamin D genetic score in the primary analysis; †SNPs used for the 122 SNP version of the vitamin D genetic score in the sensitivity analysis. INDEL: insertion and deletion; MAF: minor allele frequency.

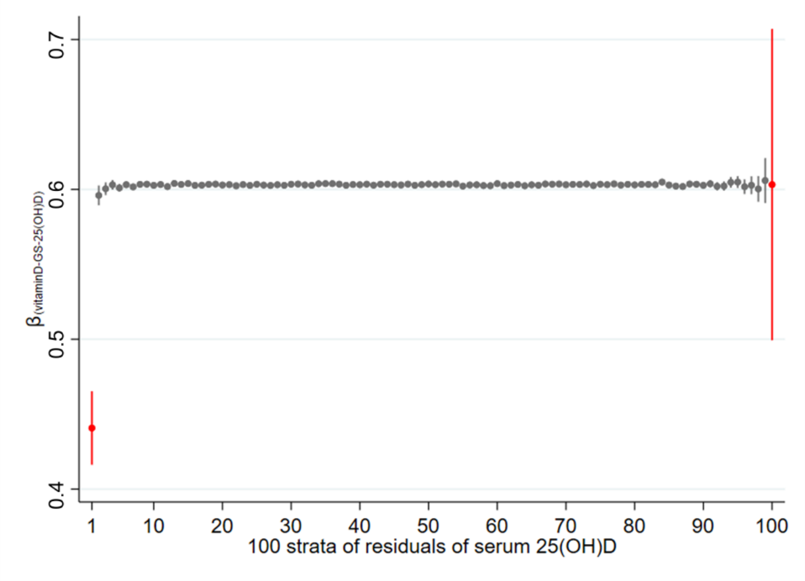

Figure S9. Distribution of the GRS-measured 25(OH)D association across 100 strata of residual measured 25(OH)D. Vitamin D-GS: vitamin D genetic score

Table S1. Association of the vitamin D genetic score with potential confounders in the UK Biobank.

	N (%)	Vitamin D-GS
		Mean (S.D.)
BMI		
Lowest 25% - 12.1-24.1	76,640 (24.9)	38.41 (5.87)
Middle 50% - 24.1-29.8	153,321 (50.0)	38.42 (5.88)
Highest 25% - 29.8-74.7	76,675 (25.0)	38.45 (5.86)
P[1]		0.88
Smoking		
Non-smokers	167,703 (54.7)	38.42 (5.86)
Ex-smokers	108,118 (35.3)	38.44 (5.89)
Current smokers	30,719 (10.0)	38.39 (5.89)
P[1]		0.18
Alcohol intake		
Daily	65,542 (21.3)	38.34 (5.88)
1 to 4 times week	155,608 (50.6)	38.45 (5.87)
1 to 3 times month	34,098 (11.1)	38.39 (5.87)
Special occasion	32,179 (10.5)	38.36 (5.90)
Never	19,963 (6.5)	38.51 (5.85)
P[1]		0.02
Physical activity		
Low	92,012 (30.6)	38.44 (5.87)
Moderate	149,205 (49.6)	38.42 (5.88)
High	59,561 (19.8)	38.42 (5.87)
P[1]		0.67
Education		
None	52,193 (17.1)	38.44 (5.86)
NVQ/CSE/A-levels	109,099 (35.8)	38.44 (5.88)
Degree/professional	143,735 (47.1)	38.42 (5.88)
P[1]		0.55
Townsend deprivation index quartiles		
Q1 lowest	76,793 (25.0)	38.41 (5.86)
Q2	76,821 (25.0)	38.46 (5.89)
Q3	76,815 (25.0)	38.42 (5.87)
Q4 highest	76,811 (25.0)	38.43 (5.88)
P[1]		0.38

NVQ, National Vocational Qualification; CSE, Certificate of Secondary Education; A-levels, Advanced levels; SD, standard deviation; Q, quartiles. Threshold for statistical significance = 0.05/6(confounders) = 0.008. P[1] Values have been adjusted for age, sex, genotyping array, birth location, and assessment center. Vitamin D-GS: vitamin D genetic score

Table S2. Functional blocks used in the leave-block-out analyses.

Functional block[a]	SNP	Gene	Traits
Blood traits	rs78151190	CARMIL1 (LRRC16A)	Various, incl. hemoglobin, platelet count, reticulocyte count
	rs1352846	GC	White cell count, granulocyte count
	rs1047891	CPS1	Various, incl. amino acid levels, platelet count, hemoglobin, metabolite levels
	rs72997623	DGAT2	Hemoglobin
	rs7528419	CELSR2	Blood protein levels, progranulin levels
	rs6672758	GALNT2	Platelet count, red cell distribution
Lipids/Metabolic	rs76798800	DCST2	Various, incl. fat-free mass, birth weight, height
	rs78151190	CARMIL1 (LRRC16A)	HbA1c, pulse, blood pressure
	rs77924615	PDILT	Blood pressure
	rs261291	ALDH1A2	Cholesterol, lipid metabolism
	rs727857	LINC01122	Fat mass, BMI, impedance, weight
	rs1047891	CPS1	Various, incl. weight, impedance, fat- free mass cholesterol
	rs12794714	CYP2R1	Hip circumference
	rs72997623	DGAT2	HDL cholesterol
	rs7528419	CELSR2	Angina pectoris, coronary artery disease, cholesterol, statin use
	rs75741381	VGF	Impedance
	rs6782190	CADM2	Fat free mass, BMI, impedance
Renal	rs77924615	PDILT	Glomerular filtration rate
	rs1047891	CPS1	Creatinine, chronic kidney disease
	rs17216707	CYP24A1	Creatinine, glomerular filtration rate
Unclassified	rs6782190	CADM2	Nervous feelings, alcohol intake, smoking, risk taking, number of children fathered
	rs61891388	RP11-867G23.13	Qualifications
	rs12803256	AP002387.1	Population differentiation
	rs1047891	CPS1	Headache
	rs212100	SULT2A1	Cholelithiasis, dehydroepiandrosterone sulphate
	rs7522116	FOXO6	Intelligence, qualifications

rs78151190	CARMIL1 (LRRC16A)	Disorders of mineral metabolism
rs261291	ALDH1A2	Age-related macular degeneration
rs12056768	LINC00536	Hair or balding pattern: pattern 4
rs1933064	FLG-AS1	Atopic dermatitis

[a]Functional blocks identified using trait associations identified through PhenoScanner V2[16].

Table S3. 122 SNP genetic score and leave block out analysis.

Vitamin D genetic score / leave-out block	SNPs excluded from the 35 SNP genetic score	Grip strength, Beta (95% CI)	P Value	Skeletal Muscle Mass, Beta (95% CI)	P Value
**122 SNP Genetic score**	**N/A**	0.01 (0.05, 0.16)	0.0004	0.004 (-0.003, 0.01)	0.24
**35 SNP Genetic score**	**N/A**	0.11 (0.04, 0.19)	0.002	0.01 (0.003, 0.02)	0.009
**Blood traits block**	rs1047891, rs1352846, rs6672758, rs72997623, rs7528419,rs78151190	0.17 (0.06, 0.27)	0.002	0.01 (0.002, 0.03)	0.0001
**Lipids / Metabolic block**	rs76798800, rs78151190, rs77924615, rs261291, rs727857, rs1047891, rs12794714, rs72997623, rs7528419, rs7574138, rs6782190	0.12 (0.04, 0.20)	0.002	0.01 (0.005, 0.02)	0.004
**Renal block**	rs1047891, rs17216707, rs77924615	0.12 (0.05, 0.19)	0.001	0.01 (0.003, 0.02)	0.007
**Unclassified block**	rs6782190, rs61891388, rs12803256, rs1047891, rs212100, rs7522116, rs78151190, rs261291, rs12056768, rs1933064	0.10 (0.02, 0.18)	0.01	0.01 (0.003, 0.02)	0.01
**Renal or Metabolic block**	rs76798800, rs78151190, rs77924615, rs261291, rs727857, rs1047891, rs12794714, rs72997623, rs7528419, rs75741381, rs6782190, rs77924615	0.12 (0.05, 0.20)	0.002	0.01 (0.002, 0.02)	0.02

Adjustment includes age, sex, assessment center, birth location, SNP array, top 40 genetic principal components, and nuisance factors which could affect serum 25(OH)D measurements, including month in which blood sample was taken, fasting time before blood sample was taken, and sample aliquots for measurement.

Table S4. Demographic characteristics of UK Biobank participants – including height and waist circumference.

		25(OH)D	25(OH)D <25 nmol/L	Grip strength[a]	Probable sarcopenia	Sarcopenic obesity	Arm skeletal muscle mass[b]
		N= 307,281	*N*=36,009	*N*=306,967[c]	*N*=25,414	*N*=16,520	*N*=302,112 [d]
	N(%)	Mean (SD)	%	Mean (SD)	%	%	Mean (SD)
**All**	307,281	49.82 (20.96)	11.70	31.04 (11.03)	8.28	5.54	5.51 (1.58)
**Sex**							
Men	144,538 (47.4)	49.86 (21.03)	11.60	39.61 (8.74)	6.39	3.98	6.92 (1.09)
Women	162,743 (53.0)	49.78 (20.89)	11.80	23.43 (6.22)	9.96	6.94	4.26 (0.60)
**Age**							
<60	169,594 (55.2)	48.38 (21.12)	13.46	32.48 (11.18)	5.46	3.40	5.56 (1.66)
≥60	137,687 (44.8)	51.57 (20.62)	9.54	29.28 (10.58)	11.75	8.23	5.44 (1.48)
**Height**							
Low, 75-163.9 cm	94,144 (30.67)	49.60 (21.00)	12.11	22.54 (6.41)	13.31	9.27	4.14 (0.65)
Mid, 164-172.9 cm	102,958 (33.54)	50.00 (21.00)	11.62	29.46 (9.02)	7.66	5.25	5.17 (1.15)
High, 173-209 cm	109,843 (35.79)	49.89 (21.00)	11.38	39.84 (9.31)	4.44	2.70	7.00 (1.21)
Missing	336 (0.11)	41.01 (21.00)	26.49	23.52 (13.70)	45.51	9.90	5.34 (1.48)
**Waist Circumference**							
Low, 20-83.9 cm	99,707 (32.45)	53.02 (21.67)	9.52	25.96 (8.27)	7.90	2.80	4.24 (0.83)
Mid, 84-95.9 cm	103,030 (33.53)	50.64 (20.75)	10.50	32.22 (11.22)	7.83	5.51	5.51 (1.26)
High, 96-197 cm	104,350 (33.96)	45.96 (19.82)	14.95	34.76 (11.33)	9.02	8.09	6.73 (1.45)
Missing	194 (0.06)	39.97 (21.33)	29.90	23.28 (13.05)	38.34	4.03	5.90 (1.54)
**BMI**							
Low 25%, 12.1-24.0	76,633 (24.9)	53.04 (22.09)	10.19	28.62 (9.62)	7.61	1.21	4.53 (1.13)
Mid 50%, 24.1-29.8	153,312 (50.0)	50.95 (20.68)	10.05	32.19 (11.17)	7.55	5.46	5.57 (1.44)
High 25% 29.8-74.7	76,667 (25.0)	44.42 (19.23)	16.40	31.23 (11.63)	10.18	9.77	6.36 (1.71)
Missing	669 (0.2)	40.69 (21.11)	26.01	25.51 (12.86)	33.73	4.33	5.34 (1.48)
**Location[e]**							
South, ≤51° Lat	102,226 (33.3)	51.43 (20.49)	9.29	30.77 (10.84)	8.35	5.20	5.53 (1.58)
Mid, 52°-53° Lat	144,470 (47.0)	49.93 (20.88)	11.35	31.10 (11.11)	8.42	5.80	5.51 (1.58)
North, 54°- ≥55° Lat.	60,585 (19.7)	46.82 (21.59)	16.62	31.36 (11.16)	7.83	2.49	5.45 (1.58)
**Smoking**							
Non-smokers	167,537 (54.5)	50.04 (20.63)	10.92	30.48 (11.00)	8.07	5.22	5.36 (1.55)
Ex-smokers	108,015 (35.2)	50.77 (21.04)	10.66	31.61 (10.99)	8.37	6.05	5.69 (1.60)
Current smokers	30,673 (10.0)	45.22 (21.80)	19.63	32.23 (11.17)	8.91	5.31	5.66 (1.60)
Missing	1,056 (0.3)	50.17 (21.77)	12.41	28.95 (11.14)	14.58	11.22	5.57 (1.60)
**Alcohol**							
Daily	65,476 (21.3)	51.22 (21.51)	11.03	33.09 (10.82)	6.51	3.93	5.71 (1.56)
1 to 4 times wk	155,474 (50.6)	50.87 (20.80)	10.19	31.85 (11.01)	7.11	4.67	5.58 (1.60)
1 to 3 times mo	34,061 (11.1)	48.08 (20.26)	12.91	29.56 (10.73)	8.46	5.85	5.35 (1.58)
Special occasion	32,125 (10.5)	46.18 (20.40)	15.74	27.05 (10.43)	12.85	9.37	5.13 (1.48)
Never	19,934 (6.5)	45.91 (20.95)	17.06	27.07 (10.66)	15.47	11.11	5.19 (1.45)
Missing	211 (0.07)	44.97 (21.05)	16.59	28.54 (11.32)	17.54	13.86	5.39 (1.57)
**Physical activity**							
Low	91,911 (29.9)	46.30 (20.20)	15.18	29.78 (10.96)	10.39	7.49	5.47 (1.59)
Moderate	149,064 (48.5)	50.60 (20.80)	10.50	31.31 (10.73)	7.17	4.65	5.47 (1.57)
High	59,518 (19.4)	54.01 (21.41)	8.11	32.65 (11.09)	6.37	3.78	5.62 (1.60)
Missing	6,788 (2.2)	43.33 (21.32)	22.48	28.32 (12.19)	20.78	14.80	5.77 (1.70)
**Education**							
None	52,119 (17.0)	50.40 (21.43)	11.89	28.56 (10.98)	14.47	10.74	5.43 (1.55)
NVQ/CSE/A-Lev.	109,007 (35.5)	50.55 (21.19)	11.22	31.02 (11.18)	7.90	5.33	5.51 (1.61)
Deg./professional	143,586 (46.7)	49.04 (20.57)	12.00	31.99 (10.79)	6.23	3.79	5.54 (1.57)
Missing	2,569 (0.84)	50.33 (20.90)	11.60	29.48 (11.22)	13.36	9.04	5.52 (1.59)
**Townsend index**							
Q1 Deprivation, least	76,746 (25.0)	51.91 (20.71)	9.16	31.71 (11.06)	6.60	4.21	5.50 (1.57)
Q2	76,745 (25.0)	51.50 (20.70)	9.35	31.31 (11.10)	7.37	4.82	5.49 (1.58)
Q3	76,719 (25.0)	49.91 (20.79)	11.20	30.92 (10.98)	8.25	5.55	5.50 (1.59)
Q4 Deprivation, most	76,711 (25.0)	45.94 (21.08)	17.11	30.24 (10.93)	10.90	7.62	5.54 (1.59)
Missing	360 (0.1)	50.02 (20.53)	11.39	31.37 (11.16)	7.22	4.84	5.61 (1.66)

Kg, kilograms; BMI, body mass index; NVQ, National Vocational Qualification; CSE, Certificate of Secondary Education; A-levels, Advanced levels; Q, quartiles; SD, standard deviation. [a] Average grip strength, min 0kg to max 85kg of contractile force, [b] Bioimpedance analysis derived skeletal muscle mass of the combined arm, min 2kg to 35.9 kg in weight, [c] Missing n= 314 (0.10%), [d] Missing n= 5,169 (1.68%). [e]Location categorization derived from assessment centre aggregation by longitudinal zoning, as described in supplementary methods (page 4). P values for all models <0.01; all models were adjusted for sex, age, assessment centre, and nuisance factors which could affect serum 25(OH)D measurements, including month in which blood sample was taken, fasting time before blood sample was taken, and sample aliquots for measurement.

Table S5. Genome-wide significant vitamin D variants used for the genetic instruments for measured 25(OH)D concentrations.

No	SNP	CHR	BP	Gene	A1	A2	A1F	UK Biobank*			SUNLIGHT Consortium[†]			SNPs for Vitamin D Genetic Score	SNPs for 122 SNP version of the Vitamin D Genetic Score
								**Beta**	**SE**	**P**	**Beta**	**SE**	**P**		
1	rs6671730[a]	1	2339139	*PEX10*	G	A	0.565714	0.0147881	0.00201077	1.92E-13	0.0061	0.0023	0.006652	Yes	Yes
2	rs35408430	1	17560195	*PADI1*	C	T	0.657806	0.0214952	0.00209979	1.36E-24	0.0236985	0.00564768	0.00002715	Yes	Yes
3	rs7522116	1	41835685	*FOXO6*	C	T	0.433767	0.0134641	0.00202533	2.97E-11	0.0116727	0.00540416	0.03077654	Yes	Yes
4	rs7528419	1	109817192	*CELSR2*	G	A	0.224671	0.0197401	0.00238729	1.35E-16	0.0179046	0.00644566	0.0054732	Yes	Yes
5	rs1933064	1	152301576	*FLG-AS1*	A	G	0.46961	0.015731	0.00203195	9.80E-15	0.0155068	0.00539368	0.00404027	Yes	Yes
6	rs76798800	1	154994978	*DCST2*	G	T	0.733745	0.0121989	0.00225962	6.71E-08	0.0173898	0.00617041	0.00482841	Yes	Yes
7	rs6672758	1	230303512	*GALNT2*	T	C	0.800872	0.0175857	0.00250898	2.40E-12	0.0156301	0.00666121	0.01895423	Yes	Yes
8	rs727857	2	58981967	*LINC01122*	G	A	0.388511	0.0140184	0.00206152	1.05E-11	0.0109131	0.00550772	0.04754487	Yes	Yes
9	rs1047891	2	211540507	*CPS1*	C	A	0.684179	0.0152142	0.00214041	1.18E-12	0.0126572	0.00572581	0.02706743	Yes	Yes
10	rs2012736	2	234622379	*UGT1A5, UGT1A6, UGT1A7, UGT1A8, UGT1A9, UGT1A10*	C	A	0.919186	0.0483073	0.00366555	1.16E-39	0.0384413	0.01038238	0.00021344	Yes	Yes
11	rs6782190	3	85639672	*CADM2*	G	A	0.352488	0.0172156	0.00208415	1.45E-16	0.0206718	0.00562451	0.00023756	Yes	Yes
12	rs705117	4	72608115	*GC*	C	T	0.1477	0.0334179	0.00280601	1.06E-32	0.0269429	0.00744137	0.00029382	Yes	Yes
13	rs1352846	4	72617775	*GC*	A	G	0.708567	0.193471	0.00219074	0	0.2221843	0.00589771	1.40E-310	Yes	Yes
14	rs78151190	6	25619007	*CARMIL1 (LRRC16A)*	A	C	0.871284	0.0168754	0.00297406	1.39E-08	0.0187117	0.00829773	0.02413132	Yes	Yes
15	rs75741381	7	100809458	*VGF*	C	G	0.852362	0.0166065	0.00282521	4.15E-09	0.0214474	0.00736197	0.00357669	Yes	Yes
16	rs12056768	8	116988527	*LINC00536*	T	G	0.417091	0.0234029	0.00202418	6.44E-31	0.0176616	0.00545433	0.00120331	Yes	Yes
17	rs77532868	10	88081438	*GRID1*	T	C	0.054042	0.0265692	0.00440069	1.57E-09	0.0280553	0.01353113	0.03813628	Yes	Yes
18	rs12794714	11	14913575	*CYP2R1*	G	A	0.578197	0.0878964	0.00201629	0	0.0702488	0.00540376	1.22E-38	Yes	Yes
19	rs61891388	11	66079818	*RP11-867G23.13*	G	T	0.455921	0.0125532	0.00200799	4.06E-10	0.0114254	0.00538961	0.03401532	Yes	Yes
20	rs1660839	11	71094232	*AP002387.1*	A	G	0.248849	0.0292665	0.00230557	6.40E-37	0.014173	0.00623639	0.02304867	Yes	Yes
21	rs12803256	11	71132868	*AP002387.1*	G	A	0.776732	0.104243	0.00239998	0	0.0839119	0.00602549	4.39E-44	Yes	Yes
22	rs12798050[b]	11	71223256	*S100A11P3*	T	C	0.830503	0.109998	0.00264849	0	0.0348	0.0024	1.00E-47	Yes	Yes
23	rs72997623	11	75488054	*DGAT2*	A	C	0.084662	0.0276158	0.00358139	1.25E-14	0.0200157	0.00937765	0.03280964	Yes	Yes
24	rs1149605	11	76485216	*RP11-21L23.4*	C	T	0.170397	0.0220166	0.00266133	1.31E-16	0.0209786	0.0072426	0.00377288	Yes	Yes
25	rs10859995	12	96375682	*HAL*	T	C	0.417366	0.0403465	0.0020206	1.05E-88	0.036551	0.00540543	1.36E-11	Yes	Yes
26	rs8018720	14	39556185	*SEC23A*	G	C	0.176673	0.0378247	0.00260904	1.26E-47	0.040852	0.00705183	6.91E-09	Yes	Yes
27	rs261291	15	58680178	*ALDH1A2*	T	C	0.644772	0.0273653	0.00208561	2.50E-39	0.0113468	0.0056366	0.04410853	Yes	Yes
28	rs77924615	16	20392332	*PDILT*	G	A	0.806515	0.0166321	0.00255158	7.11E-11	0.0195535	0.00670691	0.00355194	Yes	Yes
29	rs212100	19	48376995	*SULT2A1*	T	C	0.164001	0.0661522	0.00269018	1.61E-133	0.0193875	0.00719712	0.00706453	Yes	Yes
30	rs10426	19	51517798	*KLK10*	A	G	0.213433	0.0256629	0.00243056	4.64E-26	0.0146449	0.0065379	0.02509092	Yes	Yes
31	rs6123359	20	52714706	*BCAS1*	G	A	0.102225	0.0341831	0.00331429	6.10E-25	0.0373636	0.00940288	0.00007078	Yes	Yes
32	rs17216707	20	52732362	*CYP24A1*	T	C	0.817316	0.0376264	0.00263713	3.47E-46	0.0646902	0.0066412	2.02E-22	Yes	Yes
33	rs2585442	20	52737123	*CYP24A1*	G	C	0.240654	0.0356675	0.00237687	6.70E-51	0.0381477	0.00635676	1.96E-09	Yes	Yes

No	SNP	CHR	BP	Gene	A1	A2	A1F	UK Biobank*			SUNLIGHT Consortium[†]			SNPs for Vitamin D Genetic Score	SNPs for 122 SNP version of the Vitamin D Genetic Score
								Beta	SE	P	Beta	SE	P		
34	rs2762943	20	52790786	*CYP24A1*	G	T	0.923071	0.0457231	0.00373798	2.10E-34	0.032534	0.01442103	0.02406994	Yes	Yes
35	rs2074735	22	31535872	*PLA2G3*	C	G	0.064096	0.0278196	0.00407045	8.23E-12	0.0213678	0.01054517	0.04273241	Yes	Yes
36	rs11591147	1	55505647	*PCSK9*	T	G	0.018041	0.0450903	0.00747745	1.64E-09	-	-	-	No	Yes
37	rs2131925	1	63025942	*DOCK7*	G	T	0.356375	0.0229402	0.0020845	3.61E-28	-	-	-	No	Yes
38	rs140371183	1	152098428	*PUDPP2 (HDHD1P2)*	G	A	0.032179	0.0870147	0.00565335	1.86E-53	-	-	-	No	Yes
39	rs12123821	1	152179152	*FLG-AS1, RP11-107M16.2*	T	C	0.047527	0.0785529	0.00467652	2.55E-63	-	-	-	No	Yes
40	rs61816761	1	152285861	*FLG-AS1,FLG*	A	G	0.015926	0.12315	0.00804767	7.35E-53	-	-	-	No	Yes
41	rs10908419	1	154567699	*ADAR*	G	A	0.510067	0.012342	0.00199206	5.81E-10	-	-	-	No	Yes
42	rs11264322	1	155087933	*Y_RNA*	G	A	0.570235	0.0093848	0.0020223	3.47E-06	-	-	-	No	Yes
43	rs10908465	1	155389688	*ASH1L*	T	C	0.267332	0.0168816	0.00224924	6.12E-14	-	-	-	No	Yes
44	rs867772	1	220972343	*MARC1*	A	G	0.315487	0.0146031	0.00215141	1.14E-11	-	-	-	No	Yes
45	rs7604788	2	21190024	*RP11-116D2.1*	T	C	0.033433	0.0336737	0.0055518	1.32E-09	-	-	-	No	Yes
46	rs541041	2	21294975	*APOB*	G	A	0.180761	0.0154585	0.00258635	2.27E-09	-	-	-	No	Yes
47	rs1260326	2	27730940	*GCKR*	C	T	0.606565	0.0206128	0.00203644	4.41E-24	-	-	-	No	Yes
48	rs11127186	2	28881407	*AC074011.2*	C	T	0.495795	0.0109023	0.00203379	8.30E-08	-	-	-	No	Yes
49	rs2710651	2	63166379	*EHBP1*	G	A	0.471877	0.0114568	0.00199623	9.51E-09	-	-	-	No	Yes
50	rs3849374	2	101443397	*NPAS2, AC092168.2*	G	C	0.821971	0.0161021	0.00261564	7.46E-10	-	-	-	No	Yes
51	rs7569755	2	118648261	*HTR5BP*	A	G	0.29058	0.01425	0.00221206	1.18E-10	-	-	-	No	Yes
52	rs13060130	3	84440527	*AC108696.1*	C	T	0.860311	0.0149893	0.00287224	1.80E-07	-	-	-	No	Yes
53	rs9861009	3	141654685	*TFDP2 (RP11-271K21.11)*	C	T	0.727515	0.0140213	0.00225294	4.86E-10	-	-	-	No	Yes
54	rs78649910	4	3482213	*DOK7*	T	A	0.893821	0.0211949	0.00325203	7.15E-11	-	-	-	No	Yes
55	rs4364259	4	15892159	*RP11-442P12.1*	A	G	0.202148	0.0159119	0.00250595	2.16E-10	-	-	-	No	Yes
56	rs4616820	4	57745481	*REST*	C	T	0.535046	0.012286	0.00201755	1.13E-09	-	-	-	No	Yes
57	rs35057908	4	69372082	*UGT2B29P*	T	A	0.431309	0.0110085	0.00202343	5.31E-08	-	-	-	No	Yes
58	rs13104260	4	70348090	*UGT2B4*	A	G	0.256938	0.0072531	0.00228347	0.00149139	-	-	-	No	Yes
59	rs11732896	4	88287993	*HSD17B11*	G	A	0.701209	0.0160047	0.00217341	1.79E-13	-	-	-	No	Yes
60	rs28364331	4	100201295	*RP11-696N14.1, ADH1A*	G	A	0.018086	0.068614	0.00747169	4.19E-20	-	-	-	No	Yes
61	rs1229984	4	100239319	*ADH1B*	T	C	0.024889	0.0450574	0.00637113	1.53E-12	-	-	-	No	Yes
62	rs10070734	5	87940026	*LINC00461*	C	T	0.709531	0.0132137	0.00219753	1.82E-09	-	-	-	No	Yes
63	rs31612	5	108996643	*AC012603.1*	T	C	0.825562	0.014528	0.00264902	4.15E-08	-	-	-	No	Yes
64	rs72834856	6	22801858	*RP1-209A6.1*	T	G	0.927936	0.0249871	0.00385103	8.67E-11	-	-	-	No	Yes
65	rs28374650	6	32623367	*HLA-DQB1*	C	T	0.756438	0.0135623	0.00232502	5.44E-09	-	-	-	No	Yes
66	rs9476310	6	57767576	*RP11-325M4.2*	T	C	0.511363	0.0117571	0.00200093	4.21E-09	-	-	-	No	Yes
67	rs9490317	6	121859499	*RNU4-76P*	C	T	0.445894	0.011051	0.00201182	3.95E-08	-	-	-	No	Yes

No	SNP	CHR	BP	Gene	A1	A2	A1F	UK Biobank*			SUNLIGHT Consortium†			SNPs for Vitamin D Genetic Score	SNPs for 122 SNP version of the Vitamin D Genetic Score
								Beta	SE	P	Beta	SE	P		
68	rs2248551	6	131924689	*MED23*	G	A	0.834778	0.0233623	0.0026823	3.04E-18	-	-	-	No	Yes
69	rs10085881	7	21577960	*DNAH11*	T	C	0.717815	0.0145575	0.00223829	7.83E-11	-	-	-	No	Yes
70	rs7784802	7	64015379	*ZNF680*	T	A	0.360991	0.0138131	0.00207209	2.62E-11	-	-	-	No	Yes
71	rs6966728	7	104618318	*LINC01004*	C	T	0.537368	0.0117532	0.00203758	8.01E-09	-	-	-	No	Yes
72	rs2346264	7	133536351	*EXOC4*	A	C	0.217315	0.0138826	0.00243596	1.21E-08	-	-	-	No	Yes
73	rs34290760	8	9185179	*RP11-115J16.1*	C	G	0.970888	0.0334615	0.00593241	1.70E-08	-	-	-	No	Yes
74	rs804281	8	11611865	*GATA4*	G	A	0.583605	0.0132996	0.00202139	4.72E-11	-	-	-	No	Yes
75	rs28692966	8	25892919	*EBF2*	A	G	0.252936	0.0148311	0.00230018	1.14E-10	-	-	-	No	Yes
76	rs2725371	8	30854033	*PURG*	G	A	0.697691	0.0118392	0.00218287	5.84E-08	-	-	-	No	Yes
77	rs4738684	8	59393273	*CYP7A1*	G	A	0.665522	0.0124102	0.00211491	4.41E-09	-	-	-	No	Yes
78	rs13284054	9	107669073	*ABCA1*	C	T	0.117727	0.0175688	0.00313411	2.07E-08	-	-	-	No	Yes
79	rs10887718	10	82042624	*MAT1A*	C	T	0.471787	0.0111219	0.00199828	2.61E-08	-	-	-	No	Yes
80	rs3925446	10	91495322	*KIF20B*	A	G	0.199129	0.0152166	0.00249607	1.09E-09	-	-	-	No	Yes
81	rs4418728	10	94839724	*CYP26A1*	T	G	0.451688	0.0109611	0.00200006	4.24E-08	-	-	-	No	Yes
82	rs61883501	11	13882754	*RP11-98J9.2, RP11-98J9.3*	A	C	0.966768	0.0017709	0.00553993	0.749232	-	-	-	No	Yes
83	rs116970203	11	14876718	*PDE3B*	G	A	0.972844	0.376873	0.00612043	0	-	-	-	No	Yes
84	rs117576073	11	14912573	*CYP2R1*	G	T	0.987265	0.147177	0.00886081	5.91E-62	-	-	-	No	Yes
85	rs78168201	11	70971149	*SHANK2*	T	C	0.013766	0.0893469	0.00864963	5.18E-25	-	-	-	No	Yes
86	rs964184	11	116648917	*ZPR1 (ZNF259)*	C	G	0.868376	0.0431755	0.00294423	1.09E-48	-	-	-	No	Yes
87	rs613808	11	116710968	*APOA1-AS*	G	A	0.719993	0.0264476	0.00223913	3.40E-32	-	-	-	No	Yes
88	rs2847500	11	120114421	*POU2F3*	G	A	0.876497	0.021925	0.00302751	4.42E-13	-	-	-	No	Yes
89	rs12317268	12	21352541	*SLCO1B1*	A	G	0.848996	0.0208967	0.00278474	6.19E-14	-	-	-	No	Yes
90	rs11182428	12	38526387	*RNA5SP358*	T	C	0.480005	0.0125352	0.00199379	3.23E-10	-	-	-	No	Yes
91	rs1038165	12	68665940	*MDM1*	T	C	0.583349	0.0120567	0.002018	2.31E-09	-	-	-	No	Yes
92	rs11108368	12	96386138	*HAL*	G	A	0.606145	0.0038307	0.00209129	0.0669916	-	-	-	No	Yes
93	rs12372115	12	97982701	*RMST*	G	T	0.929281	0.0217954	0.00387948	1.93E-08	-	-	-	No	Yes
94	rs73413596	12	111582630	*CUX2*	C	T	0.073854	0.0216996	0.00382584	1.41E-08	-	-	-	No	Yes
95	rs7149014	14	29802911	*RP11-562L8.1*	T	C	0.370807	0.0129475	0.00208615	5.42E-10	-	-	-	No	Yes
96	rs12881545	14	101176212	*DLK1*	C	G	0.673439	0.011822	0.00213142	2.91E-08	-	-	-	No	Yes
97	rs1800588	15	58723675	*ALDH1A2, LIPC*	C	T	0.784797	0.0329215	0.00242187	4.38E-42	-	-	-	No	Yes
98	rs55829990	15	63790642	*USP3*	T	C	0.655996	0.0186013	0.00210294	9.12E-19	-	-	-	No	Yes
99	rs62007299	15	77711719	*PEAK1*	G	A	0.287463	0.0133407	0.00219977	1.32E-09	-	-	-	No	Yes
100	rs325384	15	100229761	*MEF2A*	C	T	0.715795	0.0141728	0.00221789	1.66E-10	-	-	-	No	Yes
101	rs17231506	16	56994528	*CETP*	C	T	0.676894	0.0184236	0.00213148	5.45E-18	-	-	-	No	Yes

No	SNP	CHR	BP	Gene	A1	A2	A1F	UK Biobank*			SUNLIGHT Consortium†			SNPs for Vitamin D Genetic Score	SNPs for 122 SNP version of the Vitamin D Genetic Score
								Beta	SE	P	Beta	SE	P		
102	rs11076175	16	57006378	*CETP*	G	A	0.178358	0.0230493	0.00260705	9.47E-19	-	-	-	No	Yes
103	rs4327060	16	72807438	*RP5-991G20.1*	C	T	0.945604	0.0243589	0.00439221	2.92E-08	-	-	-	No	Yes
104	rs4575545	16	79755446	*RP11-345M22.1, RP11-345M22.2*	G	A	0.695172	0.0155823	0.00217312	7.47E-13	-	-	-	No	Yes
105	rs11542462	16	82033810	*SDR42E1*	G	A	0.865656	0.023334	0.00291776	1.27E-15	-	-	-	No	Yes
106	rs10454087	17	40735641	*RETREG3 (FAM134C)*	C	T	0.715178	0.0135306	0.00220667	8.70E-10	-	-	-	No	Yes
107	rs2952289	17	66464414	*RP11-120M18.2*	T	C	0.798032	0.017715	0.00249217	1.18E-12	-	-	-	No	Yes
108	rs8091117	18	28919794	*DSG1*	C	A	0.934702	0.0263626	0.0040284	5.98E-11	-	-	-	No	Yes
109	rs4121823	18	47144223	*LIPG*	T	A	0.154667	0.0192879	0.00277797	3.83E-12	-	-	-	No	Yes
110	rs590215	18	57904088	*RP11-795H16.2*	C	T	0.734073	0.0129217	0.00225753	1.04E-08	-	-	-	No	Yes
111	rs2037511	18	61366207	*SERPINB11*	A	G	0.166007	0.0181228	0.00267963	1.35E-11	-	-	-	No	Yes
112	rs142158911	19	11190534	*LDLR*	A	G	0.114608	0.0255317	0.00314553	4.79E-16	-	-	-	No	Yes
113	rs187429064	19	19380513	*AC138430.4, TM6SF2*	G	A	0.011266	0.0648324	0.00947915	7.95E-12	-	-	-	No	Yes
114	rs3814995	19	36342212	*NPHS1*	C	T	0.688405	0.012558	0.00214992	5.18E-09	-	-	-	No	Yes
115	rs7412	19	45412079	*APOE*	T	C	0.082073	0.0300485	0.00363434	1.36E-16	-	-	-	No	Yes
116	rs484195	19	45421877	*APOC1*	A	G	0.384386	0.0155156	0.00209699	1.37E-13	-	-	-	No	Yes
117	rs8113404	19	53065579	*ZNF808, ZNF701*	T	C	0.304586	0.012173	0.00217136	2.07E-08	-	-	-	No	Yes
118	rs11606	19	54658102	*CNOT3*	G	C	0.425162	0.0120363	0.00205119	4.41E-09	-	-	-	No	Yes
119	rs2207132	20	39142516	*MAFB*	G	A	0.96711	0.0345955	0.00557773	5.56E-10	-	-	-	No	Yes
120	rs2229742	21	16339172	*NRIP1*	G	C	0.896549	0.0251483	0.00327069	1.48E-14	-	-	-	No	Yes
121	rs6003456	22	23356100	*AP000362.1*	T	A	0.765336	0.013277	0.00236378	1.95E-08	-	-	-	No	Yes
122	rs115621755	22	50853134	*PPP6R2*	C	T	0.67288	0.0124309	0.00212296	4.76E-09	-	-	-	No	Yes

A1: serum-25(OH)D-increasing allele; A2: alternative allele; A1F: allele frequency for A1; SE: standard error; SNP: single nucleotide polymorphism; CHR: chromosome number; BP: base-pair position, Genome Reference Consortium Human Build 37 (GRCh37); Vitamin D Genetic Score: genetic instrument (using 35 GWAS variants) for measured 25(OH)D concentration used in the primary analysis; 122 version of the Vitamin D Genetic Score: genetic instrument (using 122 GWAS variants) for measured 25(OH)D concentration used in the sensitivity analysis; *obtained from Revez, 2020 et al.[2]; †imputed summary statistics, obtained from Revez, 2020 et al.[2], serum 25(OH)D has been natural-log transformed; [a]SNP proxy in the SUNLIGHT consortium: rs1123571, r^2 = 0.86806 (1000 Genome, EUR); [b]SNP proxy in the SUNLIGHT consortium: rs2186777, r^2 = 1 (1000 Genome, EUR)

References:

1. DiaSorin. LIAISON - 25 OH Vitamin D TOTAL Assay 2019. (https://www.diasorin.com/sites/default/files/allegati_prodotti/25_oh_vit._d_total_m0870004213_e.pdf)

2. Townsend P, Phillimore P, Beattie A. Health and deprivation: inequality and the North: Routledge; 1988.

3. Vickers D, Rees P. Creating the UK National Statistics 2001 output area classification. Journal of the Royal Statistical Society. 2007;170(2):379-403.

4. Nuttall FQ. Body Mass Index. Nutrition Today. 2015;50(3):117-28.

5. Revez JA, Lin T, Qiao Z, Xue A, Holtz Y, Zhu Z, et al. Genome-wide association study identifies 143 loci associated with 25 hydroxyvitamin D concentration. Nat Commun. 2020;11(1):1647.

6. Jiang X, O'Reilly PF, Aschard HA-Ohoo, Hsu YH, Richards JBA-Ohoo, Dupuis J, et al. Genome-wide association study in 79,366 European-ancestry individuals informs the genetic architecture of 25-hydroxyvitamin D levels. Nat Commun. 2018;09(1):2041-1723.

7. Burgess S, Thompson SG. Use of allele scores as instrumental variables for Mendelian randomization. International journal of epidemiology. 2013;42(4):1134-44.

8. Palmer, T. M., J. A. Sterne, R. M. Harbord, D. A. Lawlor, N. A. Sheehan, S. Meng, R. Granell, G. D. Smith, and V. Didelez. 2011. “Instrumental Variable Estimation of Causal Risk Ratios and Causal Odds Ratios in Mendelian Randomization Analyses.” Am J Epidemiol 173 (12): 1392–1403.

9. Burgess S, Davies NM, Thompson SG. Bias due to participant overlap in two-sample Mendelian randomization. Genetic Epidemiology. 2016;40(7):597-608.

10. Burgess, S., A. Butterworth, and S. G. Thompson. 2013. “Mendelian Randomization Analysis with Multiple Genetic Variants Using Summarized Data.” Genet Epidemiol 37 (7): 658–65.

11. Bowden, J., G. Davey Smith, and S. Burgess. 2015. “Mendelian Randomization with Invalid Instruments: Effect Estimation and Bias Detection through Egger Regression.” Int J Epidemiol 44 (2): 512–25.

12. Verbanck, Marie, Chia-Yen Chen, Benjamin Neale, and Ron Do. 2018. “Detection of Widespread Horizontal Pleiotropy in Causal Relationships Inferred from Mendelian Randomization between Complex Traits and Diseases.” Nature Genetics 50 (5): 693–98.

13. Hartwig, F. P., G. Davey Smith, and J. Bowden. 2017. “Robust Inference in Summary Data Mendelian Randomization via the Zero Modal Pleiotropy Assumption.” Int J Epidemiol 46 (6): 1985–98.

14. Bowden, J., G. Davey Smith, P. C. Haycock, and S. Burgess. 2016. “Consistent Estimation in Mendelian Randomization with Some Invalid Instruments Using a Weighted Median Estimator.” Genet Epidemiol 40 (4): 304–14.

15. Kamat MA, Blackshaw JA, Young R, Surendran P, Burgess S, Danesh J, Butterworth AS, Staley JR. PhenoScanner V2: an expanded tool for searching human genotype-phenotype associations. Bioinformatics. 2019 Nov 1;35(22):4851-4853

16. Staley, James R., and Stephen Burgess. 2017. "Semiparametric Methods for Estimation of a Nonlinear Exposure-Outcome Relationship Using Instrumental Variables with Application to Mendelian Randomization." Genetic Epidemiology 41 (4): 341–52.

17. Burgess S, Mason AM, Grant AJ, Slob EA, Gkatzionis A, Zuber V, Patel A, Tian H, Liu C, Haynes WG, Hovingh GK. Using genetic association data to guide drug discovery and development: Review of methods and applications. The American Journal of Human Genetics. 2023 Feb 2;110(2):195-214.

18. Burgess, S., Davey Smith, G., Davies, N.M., Dudbridge, F., Gill, D., Glymour, M.M., Hartwig, F.P., Holmes, M.V., Minelli, C., Relton, C.L., Theodoratou, E., 2020. Guidelines for performing Mendelian randomization investigations. Wellcome Open Research 4, 186.
